# Emergence, surge, and fading of the novel feline parvovirus Thr390Ala mutant in Egyptian cats during 2023: insights from a comprehensive full-length *VP2* genetic analysis

**DOI:** 10.1186/s12917-025-05004-3

**Published:** 2025-10-03

**Authors:** Mahmoud S. Safwat, Chen Xi, Samah El-Sayed M, Ahmad Zaki Anwer, M. E. Ali, El shymaa A. Abdallah, Haitham M. Amer, Omar S. Saeed, Ghada M. Khalil, Salma W. Abdelhaleem, Reham Karam, Nehal M. Shahen, M. H. Ali, Amthal Ahmed Fouad, Mary A.N. Sargious, Samah Eid, Manar M. Farouk

**Affiliations:** 1 Department of Internal Medicine and Infectious Diseases (Infectious Diseases), Faculty of Veterinary Medicine, Cairo University, Giza, 12211 Egypt; 2 Key Laboratory of Veterinary Medicine in Universities of Sichuan Province, Southwest Minzu University, Chengdu, 610041 China; 3 Department of Internal Medicine and Infectious Diseases (Internal Medicine), Faculty of Veterinary Medicine, Cairo University, Giza, 12211 Egypt; 4 Educational Veterinary Hospital, Faculty of Veterinary Medicine, Zagazig University, Zagazig, 44511 Egypt; 5 Department of Virology, Faculty of Veterinary Medicine, Cairo University, Giza, 12211 Egypt; 6 Department of Clinical Pathology, Faculty of Veterinary Medicine, Cairo University, Giza, 12211 Egypt; 7 Department of Virology, Faculty of Veterinary Medicine, Mansoura University, Mansoura, 35511 Egypt; 8 WEQAA Central Laboratory, National Centre for the Prevention and Control of Plant and Animal Diseases (WEQAA), Riyadh, 11454 Saudi Arabia; 9 Virology Research Department, Animal Health Research Institute (AHRI), Dokki, 12618 Giza Egypt; 10 Genome Research Unit (GRU), Animal Health Research Institute (AHRI), Dokki, 12618 Giza Egypt; 11 Department of Bacteriology, Animal Health Research Institute (AHRI), Dokki, 12618 Giza Egypt

**Keywords:** Cats, Cerebellar hypoplasia, Egypt, Feline panleukopenia, Feline parvovirus, Phylogenetic analysis, *VP2* gene

## Abstract

**Background:**

Feline parvovirus (FPV) causes feline panleukopenia (FPL) and cerebellar ataxia (CA) in cats. to date, only two complete Egyptian VP2 sequences have been available in GenBank. To investigate FPV diversity And evolution in Egypt, we generated 24 complete VP2 sequences from diseased cats during two FPV activity peaks in 2023 (January-February and November-December). Egyptian sequences were Analyzed with 967 global references to assess selection pressure and phylogenetic relationships. In silico predictions of VP2 Antigenic sites, 3D structure, and phosphorylation potential were performed to evaluate the impact of identified mutations.

**Results:**

Egyptian sequences showed 99.3–100% nt And 99.8–100% aa identity among themselves, And 98.6–100% nt And 98.4–100% aa identity with global references. The overall dN/dS ratio was 0.121, with codon 101 under positive selection. Compared to the prototype FPV-b strain (M38246), Egyptian strains had 32 mutations (3 nonsynonymous: Ala5Thr, Ile101Thr, and Thr390Ala; 29 synonymous), forming 19 nt And 3 aa sequence types. Notably, Thr390Ala was unique to Egyptian sequences and absent from all global references. Phylogenetically, Egyptian strains formed two subclades: one composed solely of sequences carrying Thr390Ala (*n* = 13), And Another including the remaining 11 sequences clustering with 19 global strains sharing the synonymous mutation C135T in addition to A927G and/or A1236G. The Thr390Ala variant predominated in the first peak (11/17, 64.7%) but declined in the second (2/7, 28.6%). Residue 390 lies within an epitope-rich region (aa 350–450) and was predicted to be a phosphorylation site. Thr390Ala caused a modest drop in epitope score, disrupted local hydrogen bonding, and abolished predicted phosphorylation.

**Conclusions:**

Beyond expanding the global dataset with the largest number of Egyptian full-length VP2 sequences to date, this study highlights the Thr390Ala mutant as a classic example of evolutionary trade-off: it emerged and predominate during the first peak, potentially as an immune escape variant, but declined in the second peak, likely due to structural constraints and competition with fitter variants. Despite strong purifying selection, this case illustrates that FPV evolution is not entirely static. This underscores the need for continuous genetic monitoring to capture viral evolution in real time and inform effective control strategies.

**Supplementary Information:**

The online version contains supplementary material available at 10.1186/s12917-025-05004-3.

## Background

Feline parvovirus (FPV) is a widely distributed, devastating pathogen of domestic cats and several wild animal species [1]. FPV is the prototype of a group of antigenically related parvoviruses belonging to the species *Protoparvovirus carnivoran 1* of the genus *Protoparvovirus* within the family *Parvoviridae* [2]. FPV is a small, non-enveloped icosahedral virus characterized by a Linear, single-stranded, positive-sense DNA genome of about 5.2 kb, encoding two structural (VP1 and VP2) and two non-structural proteins (NS1 and NS2) [3].

VP2, the Major viral capsid protein, consists of 584 amino acid (aa) residues organized into an eight-stranded anti-parallel beta-barrel core motif with four surface loops inserted between the beta strands [4]. The VP2 protein plays a key role in the pathogenicity and antigenicity of FPV by mediating receptor binding, determining tissue tropism and host range, and triggering the host immune response [5]. Mutations in the VP2 protein may significantly enhance viral infectivity, transmission, persistence, and immune evasion, potentially broadening the host range [6]. Phylogenetic analysis of the *VP2* gene can categorize FPV strains into three groups: FPV-G1, FPV-G2, and FPV-G3 [6–12].

FPV is responsible for two cat disease entities: feline panleukopenia (FPL) and cerebellar ataxia (CA) [13]. FPL is a highly contagious disease clinically characterized by the sudden onset of acute, non-hemorrhagic gastroenteritis. It predominantly affects unvaccinated or inadequately vaccinated kittens between 1.5 And 6 months of age And is associated with a low survival rate, typically ranging from 20 to 55% [14]– [15]. FPL could be diagnosed using point-of-care (PoC) fecal CPV or FPV antigen (Ag) kits [16–18] or laboratory-based techniques, such as electron microscopy, viral isolation, hemagglutination test, and conventional or real-time PCR [13]– [14].

CA is the clinical presentation of cerebellar hypoplasia, a congenital anomaly caused by FPV infection during the last three weeks of gestation or within the first two weeks after birth [15]. This non-contagious, non-progressive condition is usually compatible with a normal life expectancy, although severely affected kittens may require euthanasia due to compromised quality of life [19]. A definitive diagnosis of FPV-induced cerebellar hypoplasia typically requires post-mortem detection of viral antigens or DNA in cerebellar tissues [19].

First described in 1928, FPV continues to exert its destructive effect on cat populations across the globe, encouraging ongoing research on viral genetic diversity and evolution [1]. Given the critical functions of the VP2 protein, FPV molecular characterization studies, particularly at the full-length *VP2* gene level, could help design more appropriate control and preventive strategies. However, data from Egypt are scarce, with only two complete *VP2* sequences reported to date [20]. Therefore, the present study reports the first comprehensive genetic Analysis of Egyptian FPV strains utilizing 24 complete *VP2* sequences obtained from cats clinically diagnosed with FPL And CA alongside a global dataset of 967 reference strains.

## Materials and methods

### Study population and sampling

The study population consisted of 26 client-owned cats admitted with clinical signs suggesting FPV infection: acute gastroenteritis (*n* = 22) or CA (*n* = 4). Given that the natural peak of FPV activity in Egypt typically occurs during winter (AbdElbaky 2022), samples were submitted during two Anticipated peak periods in 2023: January–February (*n* = 17) and November–December (*n* = 7). These originated from ten private veterinary clinics located across three Egyptian governorates: Cairo, Giza, and Al-Qalyubia. The cats with acute gastroenteritis were neither littermates nor from the same household, whereas those with CA were sibling pairs from two different litters. Owners’ complaints, physical examination findings, and data regarding age, sex, breed, and FPV vaccination status were recorded. Rectal swabs were collected from all cats with acute gastroenteritis.

Of the four kittens with cerebellar ataxia, two, each from a different litter, were humanely euthanized due to the severity of their neurological impairments, which rendered them functionally incapable of performing essential activities such as feeding, grooming, or ambulating safely. The prognosis for adaptation and quality of life was assessed as extremely poor to nil, with a high risk of injury, persistent distress, and failure to thrive, raising significant ethical concerns. Additionally, the owners were unable to provide the intensive, long-term supportive care required for sustained humane management. Euthanasia was performed in accordance with the AVMA Guidelines for the Euthanasia of Animals (2020): kittens were first deeply anesthetized with ketamine (10 mg/kg IM) and xylazine (1 mg/kg IM), followed by intravenous administration of 20% sodium pentobarbital (150 mg/kg), resulting in rapid and painless death. Death was confirmed by the absence of heartbeat and respiration, as well as the loss of both pupillary and corneal reflexes. After that, cerebella were collected from both kittens. The remaining two kittens with cerebellar ataxia were not euthanized, as their neurological impairments were compatible with potential adaptation and long-term care with owner assistance. Consequently, no cerebellar tissue was collected, and no further laboratory testing, such as PCR, was performed. Nonetheless, these kittens were retained to support documentation of clinical signs and relevant epidemiological data.

Rectal swabs and cerebella were kept frozen at −20 °C at the Laboratory of Internal Medicine and Infectious Diseases Department, Faculty of Veterinary Medicine, Cairo University, until molecular investigations were performed. The current study was approved by the Institutional Animal Care and Use Committee, Cairo University (FVMCU/131020241040). Oral informed consent was obtained from the cat guardians before sample collection and data use.

### Screening samples for FPV Ag and DNA

According to the manufacturer’s protocols, rectal swabs were tested for fecal FPV Ag using specific PoC kits, namely Anigen^®^ FPV Ag (BioNote, South Korea) or VDRG^®^ FPV Ag (Median, South Korea). These kits are based on lateral flow immunochromatography technology, and samples were considered positive if two lines appeared within the result window.

Genomic DNA was isolated from rectal swabs and cerebella using the QIAamp^®^ Fast DNA Stool Mini Kit and the QIAamp^®^ DNA Mini Kit, respectively, following the manufacturer’s instructions. All samples were examined using a conventional PCR methodology that amplifies the full-length *VP2* gene using three overlapping primer pairs [21]. PCR amplification was performed in the GeneAmp 9700 thermal cycler (ThermoScientific, USA) using the DreamTaq PCR Master Mix (ThermoFisher, USA). Data concerning primers (names, sequences, and positions), expected amplicon size, And the number of amplification cycles And annealing temperature of the used conventional PCR are provided in Supplementary Table 1. To validate PCR results, FPV strain FVMCU/018 (accession no. PP663049) and nuclease-free molecular-grade PCR water were included in each PCR run as positive and negative controls, respectively.

### Genetic analysis of FPV

All positive samples were further sequenced to obtain complete FPV *VP2* gene sequences from Egyptian cats. The PCR products were purified from the agarose gel using the QIAquick^®^ Gel Extraction Kit (Qiagen, Germany), following the manufacturer’s protocols. Purified amplicons were sequenced in both directions using the same primers employed in the conventional PCR and the BigDye™ Direct Cycle Sequencing Kit (ThermoFisher, USA), according to the manufacturer’s instructions. The obtained sequences were checked for chromatogram quality, edited, And assembled using the BioEdit software version 7.0.9.1. A global dataset of reference FPV strains was created through GenBank searches (accessed in March 2025) for all FPV complete *VP2* gene sequences using the keywords “feline parvovirus,” or “feline panleukopenia virus,” or “*Protoparvovirus carnivoran 1*” or “*Carnivore protoparvovirus 1*” and “*VP2* gene” and “complete cds.” Egyptian sequences obtained in this study were aligned with reference sequences using the “ClustalW Multiple alignment” tool implemented in the BioEdit software.

The alignment was checked for recombinant events in the Recombination Detection Program 5 (RDP 5) using the following algorithms: Chimaera, GENECONV, MaxChi, RDP, SiScan, And 3Seq. A significant recombinant event was defined as one detected using three or more algorithms at *P* < 0.01. The alignment was translated into the deduced amino acid sequences in BioEdit. The identity of parvovirus was determined through a manual check of the key amino acids that discriminate between FPV and CPV-2 at positions 80 (lysine in FPV vs. arginine in CPV-2), 93 (lysine in FPV vs. asparagine in CPV-2), 103 (valine in FPV vs. alanine in CPV-2), 323 (aspartic acid in FPV vs. asparagine in CPV-2), 564 (asparagine in FPV vs. serine in CPV-2), And 568 (alanine in FPV vs. glycine in CPV-2) of the VP2 protein. Sequences exhibiting one or more CPV-2-specific amino acids at these key residues were identified as FPV-like strains. To maintain a higher degree of alignment homogeneity and focus on the evolutionary relationships among typical FPV strains, all recombinant and FPV-like strains were removed from the final dataset.

Nucleotide and amino acid identity matrices were constructed using BioEdit software to calculate the homology percentage among FPV strains at the nucleotide and amino acid levels. The selection pressure driving FPV evolution was assessed by estimating the ratio of synonymous mutations per synonymous site (dS) to nonsynonymous mutations per nonsynonymous site (dN) using the Datamonkey web server (accessed in April 2025). A dS/dN ratio of < 1, = 1, and > 1 indicates negative, neutral, and positive selection, respectively. Two models available through the Datamonkey web interface, namely Fast Unconstrained Bayesian AppRoximation (FUBAR) and Single-Likelihood Ancestor Counting (SLAC), were used to identify sites prone to pervasive selection pressure, using a posterior probability threshold of ≥ 0.9 and a *P*-value < 0.05, respectively. A third algorithm, the Mixed Effects Model of Evolution (MEME), was employed to detect sites under episodic selection pressure at a *P*-value < 0.05.

The evolutionary relationship between FPV strains was inferred by the maximum likelihood (ML) statistical method using MEGA software version 11. The Tamura-3-Parameter model with a discrete gamma distribution and allowing for invariant sites (T92 + G + I) was selected as the best nucleotide substitution model to fit our dataset, determined using the “Find Best DNA Model” tool implemented in MEGA. The reliability of tree branching orders was assessed using the bootstrap method (1000 iterations). The obtained tree was edited using the FigTree software version 1.4.4.

To account for the potential negative influence of a large number of highly similar sequences on bootstrap support values, the analysis was repeated using all sequences generated in this study alongside a smaller, representative subset of reference sequences. The maximum likelihood (ML) method was applied with the T92 + G + I nucleotide substitution model And 1,000 bootstrap replicates.

### In silico prediction of B-cell epitopes, protein structure, and phosphorylation sites in VP2

Amino acid mutations identified in the VP2 sequences of Egyptian FPV strains were analyzed to assess their potential structural and functional consequences using several bioinformatic tools. Epitope prediction was performed using BepiPred-3.0 (https://services.healthtech.dtu.dk/services/BepiPred-3.0/) to evaluate the influence of mutations on Antigenic activity. A threshold Antigenicity score of 0.150 was applied, with values above this cutoff considered indicative of linear B-cell epitopes.

Three-dimensional (3D) structural modeling of the VP2 protein was carried out using SWISS-MODEL (https://www.swissmodel.expasy.org/interactive), based on the FPV VP2 template with the highest sequence homology (PDB ID: 4qyk.1.A). Structural alignment and hydrogen bond analysis were conducted using UCSF ChimeraX software (version 1.10) to visualize the effects of specific mutations on local protein conformation and bonding interactions.

Phosphorylation site prediction was performed using NetPhos-3.1 (https://services.healthtech.dtu.dk/services/NetPhos-3.1/) to assess whether identified mutations altered the predicted phosphorylation potential of the VP2 protein.

### Mapping and spatial visualization

Geospatial Mapping was conducted to visualize the distribution of FPV-positive samples during two distinct peaks of disease activity in 2023 (January–February and November-December). Administrative boundary data for Egypt were retrieved from the Global Administrative Areas database (GADM v4.1; Level 1) and imported using the GeoPandas library (v0.14.3) within a Jupyter Notebook environment (Python v3.11.8, Anaconda distribution). Background maps for the continental and national context were sourced from the Natural Earth dataset.

Latitude and longitude coordinates of the veterinary clinics from which samples were submitted were used to georeference each location. These coordinates served as the anchor points for mapping individual sample origins. Each FPV-positive sample appeared as a color-coded circle, reflecting its mutation status. When multiple samples originated from the same clinic, minor horizontal displacement was applied to reduce symbol overlap; however, they remained centered around the clinic’s actual coordinates.

Map generation and annotation were conducted using Matplotlib (v3.8.4) and Cartopy (v0.23.0). A distance scalebar was included using the matplotlib-scalebar library (v0.8.1) and longitude/latitude axes were formatted in decimal degrees.

### Statistical analysis

To assess the association between identified mutations and disease severity, we compared disease outcomes (recovery vs. death/euthanasia) among FPV-positive cats infected with viral strains that either carried or lacked these mutations, using Fisher’s exact test.

## Results

### Definitive diagnosis of FPV infection in the study population

All cats presenting with acute gastroenteritis tested positive for fecal FPV Ag using PoC kits (22/22, 100%), confirming FPL diagnosis. Additionally, all rectal swabs from cats with acute gastroenteritis and cerebellar tissue samples from euthanized kittens with CA yielded clear amplicons with all three overlapping primer pairs, further confirming FPL and providing a clue for a definitive diagnosis of FPV-associated CA in the two litters.

### Epidemiological features and clinical findings

Cats with FPL (*n* = 22) were ≤ six-month-old, mixed-breed kittens, including 10 females And 12 Males, And none had been vaccinated against FPV. Twelve kittens were discharged alive, recording a survival rate of 54.5% (12/22), with a median hospitalization time to recovery of 7 days (ranging from 3 to 7 days). Owners’ complaints and physical examination identified variable clinical presentations in affected kittens at admission as follows: normal temperature (10/22, 45.5%), fever (10/22, 45.5%), subnormal temperature and lateral recumbency (2/22, 9%), anorexia and lethargy (22/22, 100%), vomiting (22/22, 100%), diarrhea (13/22, 59%), hemorrhagic diarrhea (2/22, 9%), and dehydration (22/22, 100%).

**Fig. 1:**
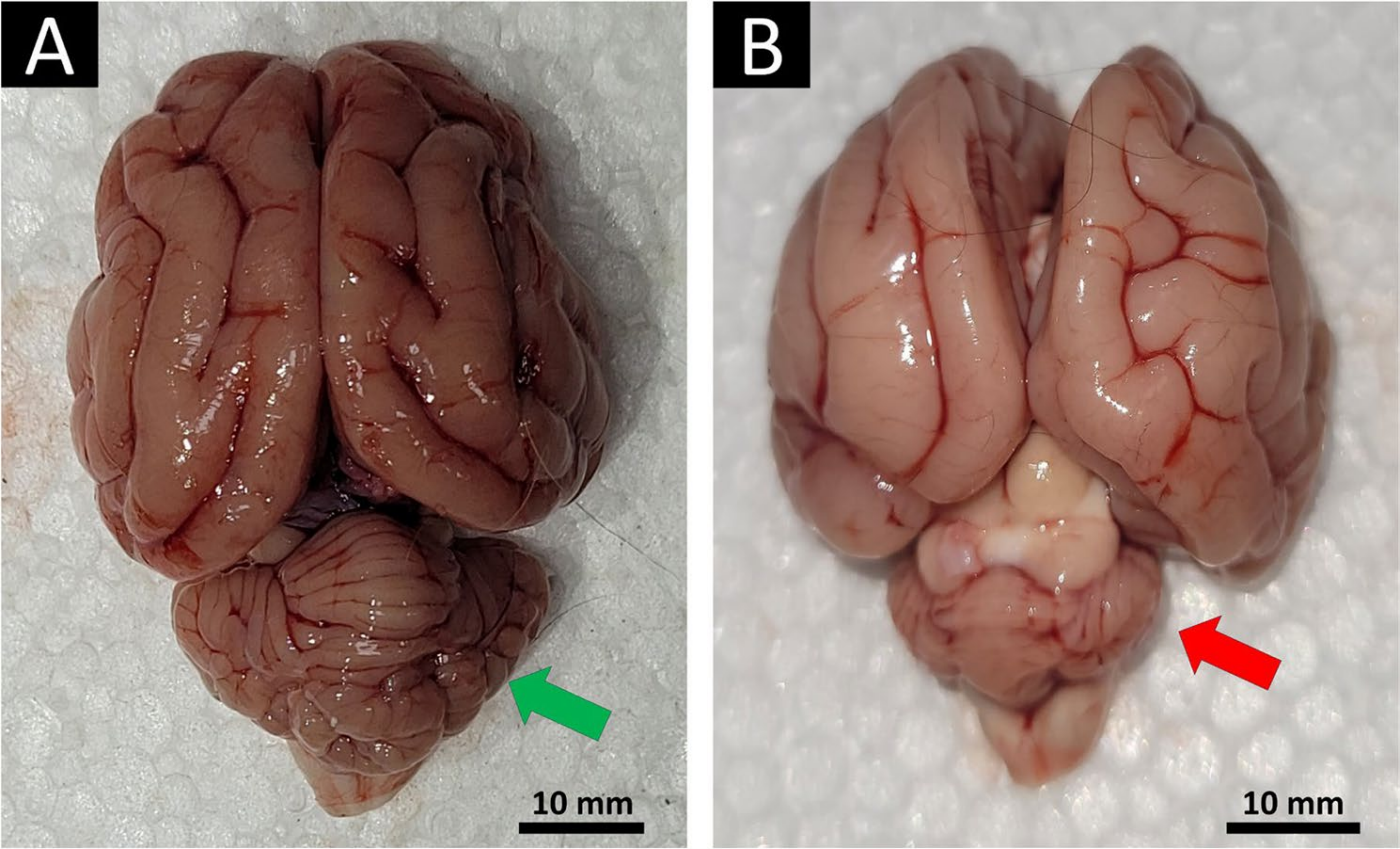
Gross brain tissues from two 8-week-old kittens presenting with acute gastroenteritis (**A**) or cerebellar ataxia (**B**). Note the markedly smaller cerebellum in the ataxic kitten (green arrow), consistent with cerebellar hypoplasia, compared to the other kitten (red arrow). An approximate 10 mm scale bar is applied to each image, estimated from the anteroposterior (AP) length of the telencephalon (i.e., the cerebral hemispheres, from frontal to occipital poles), assumed to be 39 mm at this age. This estimate is based on data showing that AP Length increases from 30.5 mm at postnatal week 2 to 36.9–38.2 mm at weeks 6–7, reaching ~ 40.7 mm in adults [22]

Cats admitted with CA (*n* = 4) were mixed-breed, one-month-old kittens, including two males and two females. They represented two pairs of siblings, each belonging to a separate litter of five kittens; the remaining three kittens in each litter were healthy. None of the litter’s dams had been vaccinated against FPV. Owners’ complaints and physical examination revealed that all affected kittens showed the following clinical signs with variable severities: wobbly gait, which was first noticed by owners at the time of ambulation, standing with a wide-based stance, fine head tremors at rest, intentional tremors, and swaying (Supplementary videos 1 And 2). Gross examination of brain tissues from the two euthanized kittens with CA revealed markedly reduced cerebellar size (cerebellar hypoplasia) compared to age-matched kittens that died of FPL (Fig. 1).

### Genetic analysis of FPV strains

Twenty-four complete *VP2* gene sequences (1755 bp) were successfully generated in the current study and deposited in GenBank under the following accession numbers: PV521942-PV521965. One thousand complete or nearly complete (1743 bp) *VP2* gene sequences were downloaded from GenBank and aligned with the Egyptian sequences.

Five recombination events were identified in the alignment, none of which involved the Egyptian sequences generated in this study. Details of the recombinant breakpoints, parental strains, And detection methods are provided in Supplementary Table 2. In addition, twenty-seven reference sequences were classified as FPV-like strains based on the presence of one or more CPV-2–specific aa at key discriminatory residues (Supplementary Table 3). All detected recombinant And FPV-like strains were excluded from the alignment, resulting in a final dataset of 991 sequences, including 24 Egyptian And 967 reference sequences.

In the final dataset, reference sequences included FPV strains collected over 60 years (1964–2023) from domestic cats (*n* = 895), domestic dogs (*n* = 25), 22 different wild animal species (*n* = 64), and commercial vaccines (*n* = 7). They were geographically distributed as follows: 14 from Africa (Egypt [*n* = 2] and Nigeria [*n* = 12]), 625 from Asia (Bangladesh [*n* = 1], China [*n* = 508], India [*n* = 11], Japan [*n* = 13], Korea [*n* = 30], Taiwan [*n* = 2], Thailand [*n* = 21], the UAE [*n* = 11], and Vietnam [*n* = 28]), 152 from Europe (Belgium [*n* = 1], Finland [*n* = 1], Germany [*n* = 1], Hungary [*n* = 4], Italy [*n* = 107], Portugal [*n* = 25], Russia [*n* = 1], Spain [*n* = 2], Turkey [*n* = 3], and the United Kingdom [*n* = 7]), 33 from North America (Canada [*n* = 11] and the USA [*n* = 22]), 124 from Oceania (Australia [*n* = 115] and New Zealand [*n* = 9]), And 12 from South America (Argentina [*n* = 9] and Brazil [*n* = 3]). Data regarding host, geographical distribution, collection date, And GenBank accession number of each reference FPV strain in the final dataset are provided in Supplementary Table 4.

FPV strains in the final dataset exhibited 98.3–100% And 97.4–100% identity at the nucleotide and amino acid Levels, respectively. The Egyptian sequences shared 99.3–100% And 99.8–100% nucleotide And amino acid identity, respectively, with each other, And 98.6–100% And 98.4–100% identity, respectively, with the reference strains.

FPV strains in the final dataset showed a dN/dS ratio of 0.121. All three models (FUBAR, SLAC, and MEME) indicated that the amino acid residue at position 101 of *VP2* was subject to positive selection pressure. Additionally, the FUBAR And SLAC models identified 221 And 61 sites under negative selection pressure, respectively.

Compared to the prototype strain FPV-b (accession no. M38246), the Egyptian sequences exhibited thirty-two variable nucleotide positions, three of which were nonsynonymous: G13A (Ala5Thr), T302C (Ile101Thr), and A1168G (Thr390Ala), observed in 4% (1/24), 100% (24/24), And 54% (13/24) of Egyptian sequences, respectively, And 1.8% (18/967), 95.6% (925/967), And 0% (0/967) of reference sequences, respectively. Data regarding reference sequences that showed Thr substitutions at position 5 or retained the prototype strain’s amino acids at position 101 are provided in Supplementary Tables 5 And 6, respectively.

Nucleotide variations from the prototype strain classified the Egyptian sequences into 19 different nucleotide sequence types (nSTs) (Table 1). None of the Egyptian nSTs showed 100% identity with any reference strains, except for nST19, which was identical to two reference sequences obtained from an Egyptian dog and a Thai cat (accession no. OM638043 and MW589472, respectively).

**Table 1:**
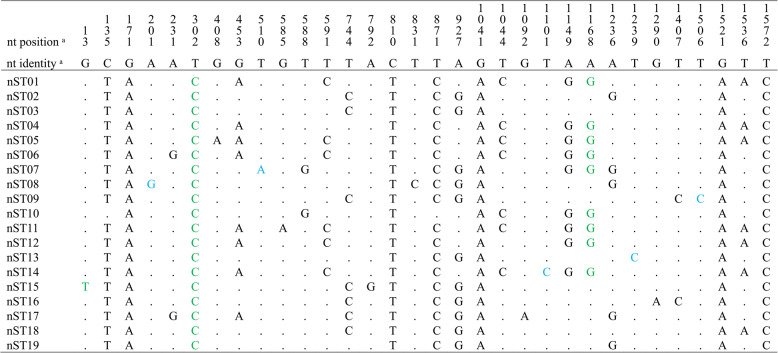
The classification of Egyptian FPV full-length VP2 gene sequences generated in this study into different nucleotide sequence types (nSTs) according to their genetic variation from the prototype strain FPV-b (GenBank acc. no M38246)

ᵃ nucleotide position and type according to FPV-b (accession no. M38246)

Egyptian sequences generated in this study represented 19 nSTs: nST01, including five sequences (GenBank acc. no PV521945, PV521957-PV521959, and PV521965), nST02, including two sequences (GenBank acc. no PV521953-PV521954), and nST03-nST19, each containing one sequence with the following GenBank acc. no: nST3-nST5 (PV521942-PV521944, respectively), nST6-nST12 (PV521946-PV521952, respectively), nST13-nST14 (PV521955-PV521956), and nST15-nST19 (PV521960-PV521964, respectively)

Dots indicate that Egyptian sequences have the same nucleotides as the prototype strain at these positions

Blue-font letters indicate that these mutations are singletons in the final dataset used in this study (991 sequences)

Green-font letters indicate the three nonsynonymous mutations detected in Egyptian sequences

At the polypeptide level, compared to the prototype strain, the Egyptian sequences represented three amino acid sequence types (aaSTs): aaST1-aaST3, exhibiting one (T302C/Ile101Thr), two (T302C/Ile101Thr and A1168G/Thr390Ala), and two (G13A/Ala5Thr and T302C/Ile101Thr) nonsynonymous mutations, respectively. The aaST1-aaST3 included 10 (accession no. PV521942, PV521948-PV521949, PV521953-PV521955, and PV521961-PV521964), 13 (accession no. PV521943-PV521947, PV521950-PV521952, PV521956-PV521959, and PV521965), and one (accession no. PV521960) Egyptian sequences, respectively. The aaST1-aaST3 were 100% identical to 351, 0, And 16 reference sequences, respectively. The amino acid sequences derived from kittens with cerebellar ataxia did not form a unique aaST; instead, they clustered within aaST1 and aaST2, alongside sequences from kittens exhibiting feline panleukopenia.

**Fig. 2:**
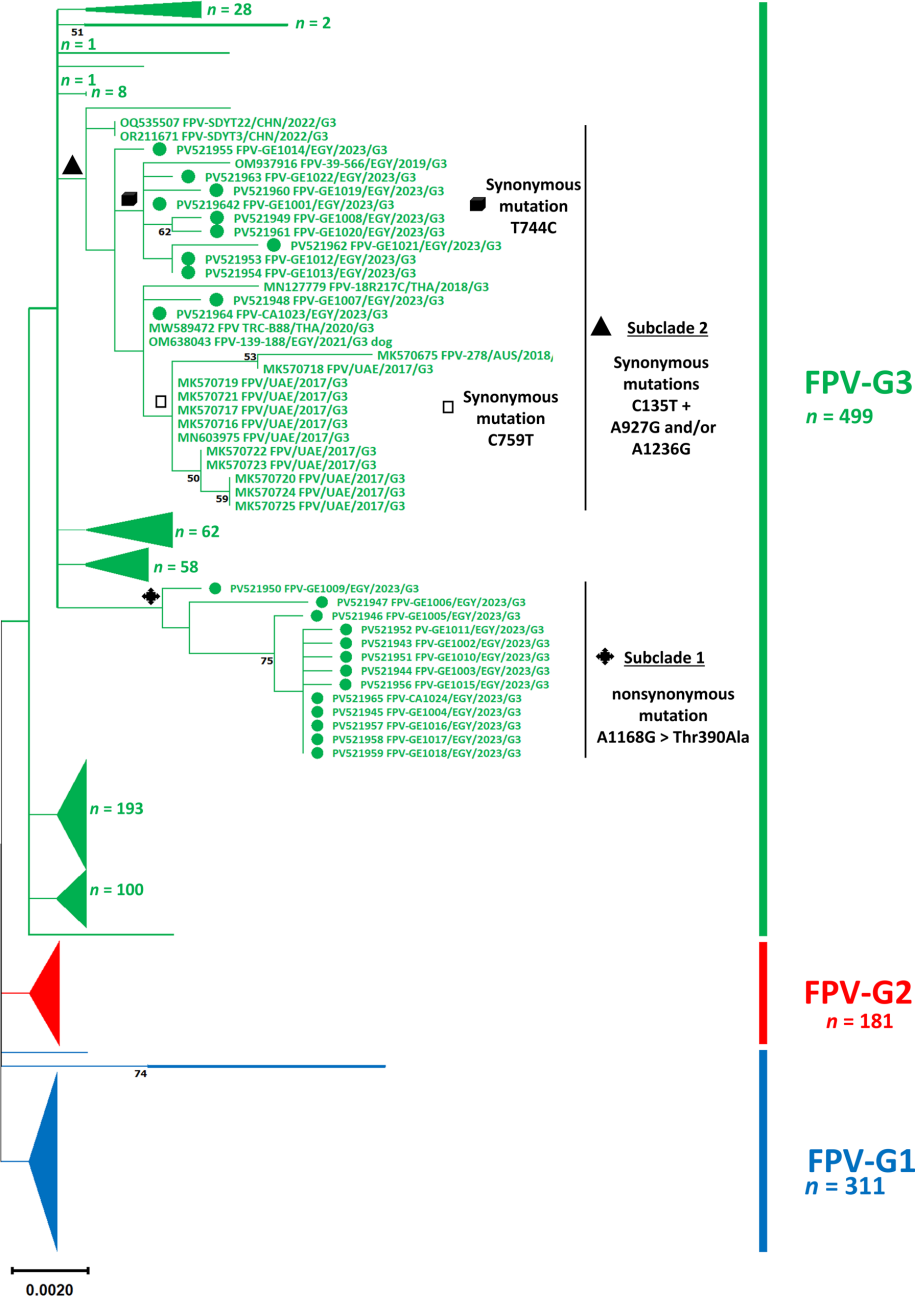
A maximum likelihood phylogenetic tree constructed from nearly full-length VP2 gene sequences (1743 bp) of 991 feline parvovirus (FPV) strains, including 24 Egyptian sequences generated in this study (highlighted with green circles) And 967 reference sequences (see Supplementary Table 4 for GenBank accession numbers). Strains clustered into three distinct groups: FPV-G1 (blue), FPV-G2 (red), and FPV-G3 (green). All Egyptian sequences belonged to FPV-G3 and formed two subclades defined by distinct mutation profiles: subclade 1 carried the nonsynonymous mutation A1168G (Thr390Ala), while subclade 2 featured shared synonymous mutations C135T, A927G, and/or A1236G. Bootstrap values > 50% are shown. The scale bar represents nucleotide substitutions per site

**Fig. 3:**
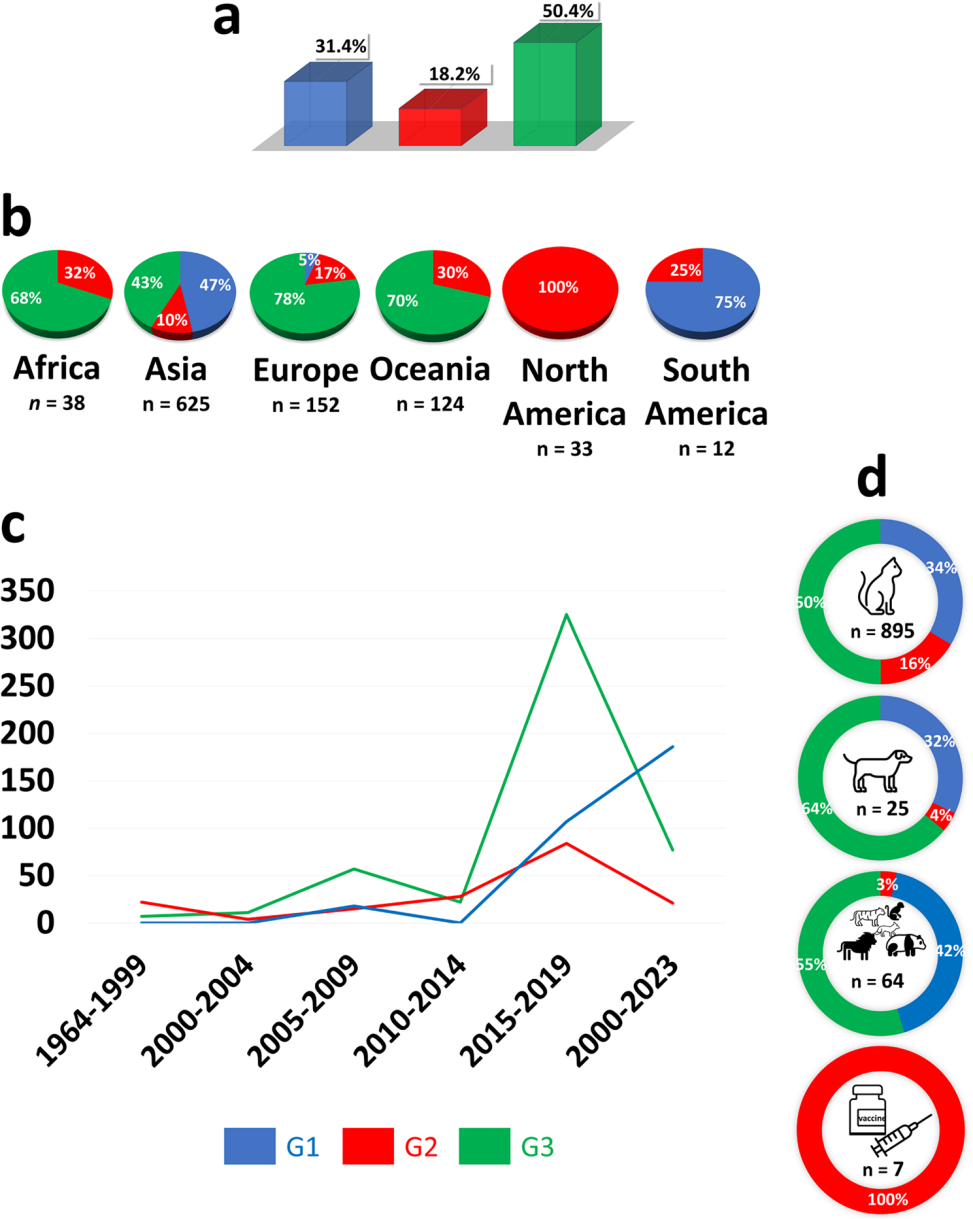
Metadata summary of FPV groups G1–G3, including overall prevalence (**a**), geographical (**b**), temporal (**c**), and host/vaccine (**d**) distribution. G3 is the dominant group globally, followed by G1, while G2 is the least represented (**a**). Geographically, G3 predominates in Africa, Europe, and Oceania; G1 in Asia and South America; and notably, G2 is the only group detected in North America (**b**). Notably, although G3 is the globally predominant group, G1 has shown recent spread and expansion (**c**). Interestingly, all commercial FPV vaccines analyzed to date belong exclusively to G2 (**d**)

The ML phylogenetic tree, constructed from a final dataset of 991 sequences (each 1743 nucleotides in length), grouped FPV strains into three clusters: FPV-G1 to FPV-G3 (Fig. 2). However, these groupings were not supported by consistent genetic markers or high bootstrap values. The tree was compressed to emphasize the evolutionary positioning of the Egyptian sequences relative to global reference strains. A full-resolution version of the tree, including detailed visualization of the three FPV groups, is provided in the Supplementary Materials, along with the corresponding FASTA alignment. Figure 3 summarizes the frequency and distribution of FPV strains by host species, geographic region, and time period within each group.

All Egyptian sequences were clustered within FPV-G3 And were divided into two distinct subclades, designated subclade 1 And subclade 2. Subclade 1 comprised 13 Egyptian sequences characterized by the unique nonsynonymous mutation A1168G/Thr390Ala. Subclade 2 included the remaining 11 Egyptian sequences along with 19 reference strains from Australia (*n* = 1), China (*n* = 2), Egypt (*n* = 2), Finland (*n* = 1), Thailand (*n* = 2), and the UAE (*n* = 11). These sequences all shared the synonymous mutation C135T and exhibited the synonymous mutations A927G and/or A1236G. Within subclade 2, additional clustering based on specific synonymous mutations was observed. One cluster included 11 Egyptian sequences (10 from this study and one previously reported), all sharing the synonymous mutation T744C, which was absent from other reference strains. Another distinct cluster comprised 13 sequences from the UAE and Australia, unified by the nonsynonymous mutation C759T.

**Fig. 4:**
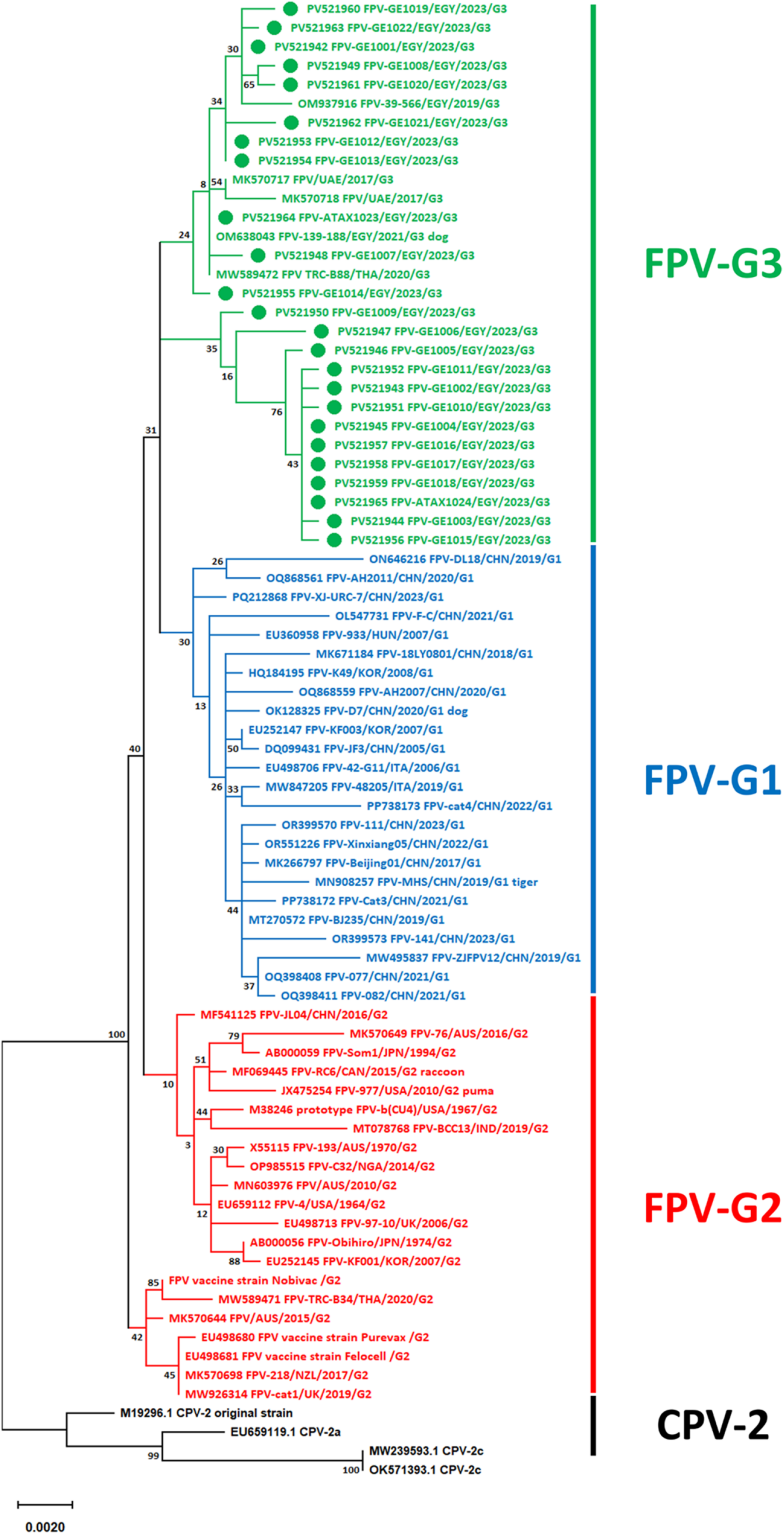
Maximum likelihood phylogenetic tree based on nearly full-length VP2 gene sequences (1743 bp), including 24 Egyptian sequences generated in this study (indicated by green circles) and a representative subset of reference strains (*n* = 54). The tree is rooted using CPV-2 strains. Despite data curation and reduced taxon sampling, bootstrap support at key group nodes remained low, and both G2 and G3 appeared polyphyletic. Egyptian sequences (subclades 1 And 2) retained their topological positions and bootstrap values, consistent with those observed in the full dataset. The scale bar indicates the number of nucleotide substitutions per site

When the ML analysis was repeated using our sequences alongside a smaller, representative subset of reference strains, the low bootstrap values persisted, and both G2 and G3 strains appeared polyphyletic. Despite this, the Egyptian sequences consistently clustered into the same subclades with identical topological structure and bootstrap support (Fig. 4).

### Structural, antigenic, and phosphorylation impacts of VP2 amino acid substitutions

**Fig. 5:**
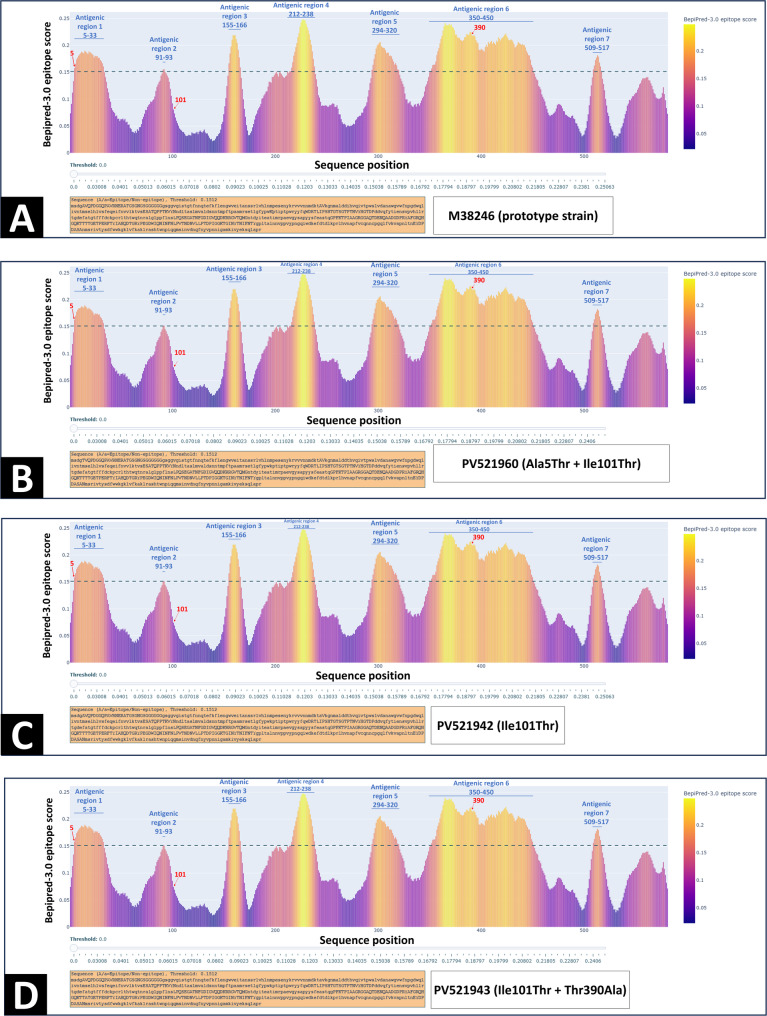
Linear B-cell epitope predictions for the VP2 protein of feline parvovirus (FPV) using the BepiPred 2.0 algorithm. The amino acid sequence of the prototype FPV strain (accession no. M38246) (**A**) was used as the reference. Seven predicted antigenic regions—5–33, 91–93, 155–166, 212–238, 294–320, 350–450, And 509–517—are indicated by blue horizontal lines and labels. Three mutation-associated sites with altered epitope scores are marked with red arrows: Ala5Thr, Ile101Thr, and Thr390Ala. The Ala5Thr mutation increased the epitope score (**B**). The Ile101Thr mutation reduced the score at positions 91–92 And abolished the predicted epitope status of position 93 (**B**, **C**, **D**). The Thr390Ala mutation decreased the epitope score at position 390 (**D**). The dashed line indicates the threshold for predicted epitope residues (score = 0.15)

Comparative epitope prediction of VP2 amino acid sequences from wild-type (M38246) and representative mutant strains (PV521942 for Ile101Thr; PV521943 for Ile101Thr + Thr390Ala; PV521960 for Ala5Thr + Ile101Thr) identified seven conserved Antigenic regions across all strains: 5–33, 91–93, 155–166, 212–238, 294–320, 350–450, And 509–517 (Fig. 5). Residue 5 (within epitope 5–33) And residue 390 (within epitope 350–450) exhibited altered antigenicity upon mutation: Ala5Thr increased the epitope score (0.158 → 0.163), whereas Thr390Ala decreased it (0.225 → 0.221). Although residue 101 is not part of a defined epitope, the Ile101Thr substitution reduced the average epitope score of region 91–92 (0.155 → 0.151) And eliminated the epitope status of position 93 (0.151 → 0.145), falling below the threshold of 0.15. Figure 5 also depicts the antigenicity curves across all five strains, highlighting mutation-induced shifts in predicted reactivity.

**Fig. 6:**
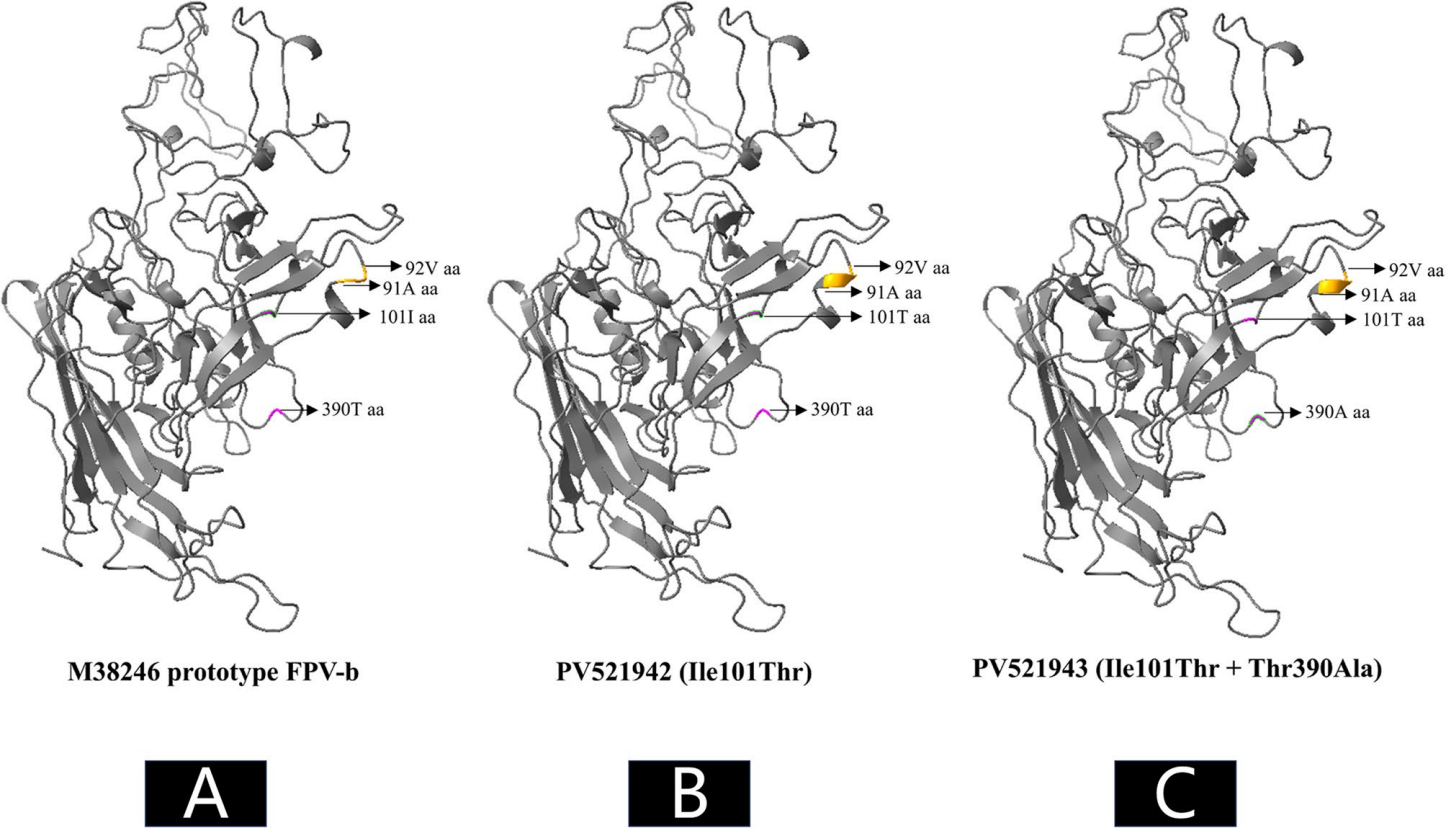
In silico prediction of 3D structural changes in the FPV VP2 protein caused by mutations identified in Egyptian sequences. Compared to the prototype strain (**A**), the Ile101Thr mutation altered the conformation of residues 91–92 from a random coil to an α-helix (**B**), whereas the Thr390Ala mutation did not affect the overall 3D structure of the VP2 protein (**C**)

**Fig. 7:**
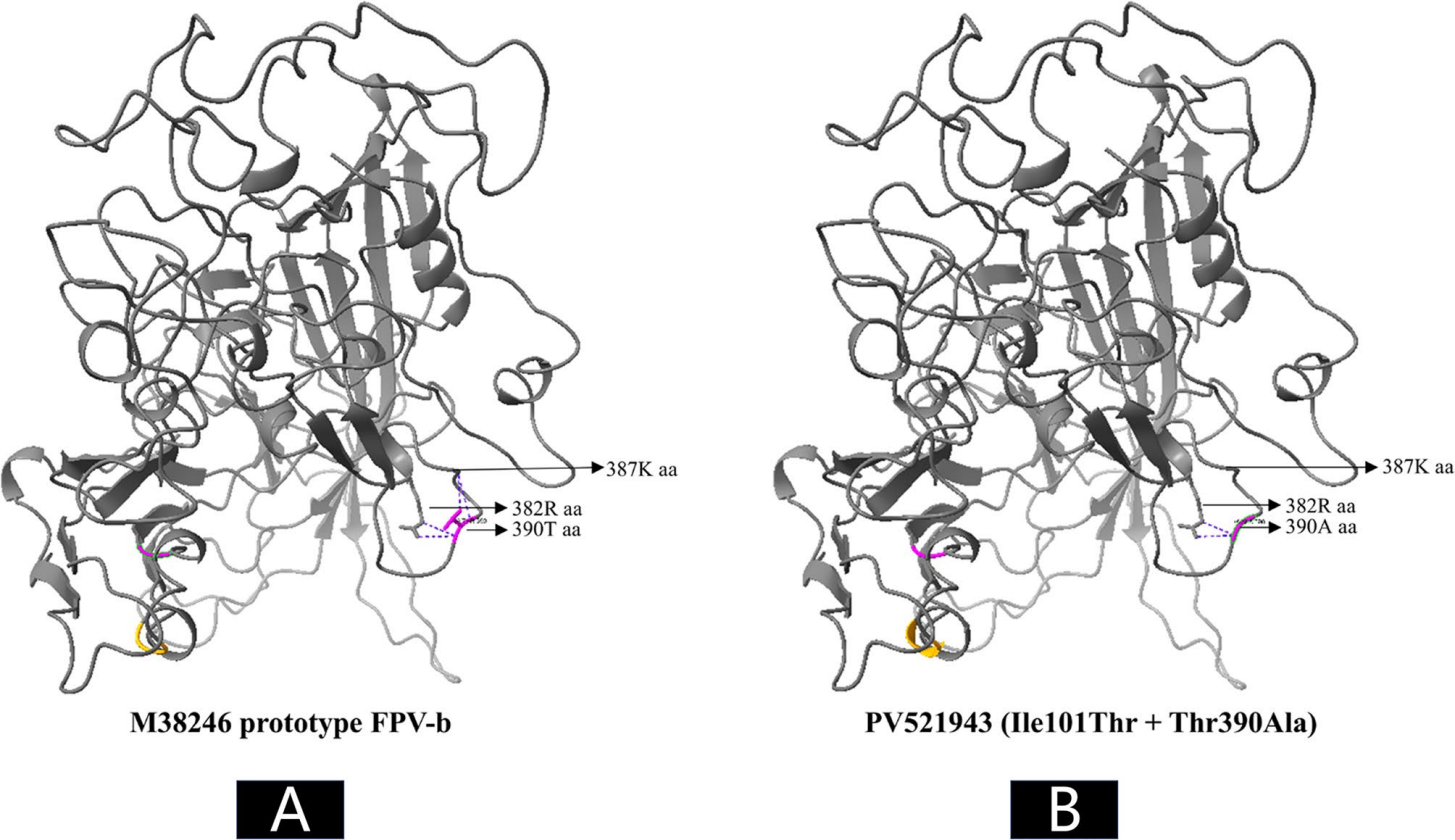
In silico prediction of the effect of mutations identified in Egyptian FPV sequences on hydrogen bonding potential. In the prototype strain, threonine at position 390 forms four hydrogen bonds with residue 382 And two with residue 387 (**A**). Substitution with alanine at this position Leads to the loss of hydrogen bonds with residue 387 And a reduction to two bonds with residue 382 (**B**)

Structural alignment revealed that Ile101Thr significantly altered the local conformation (residues 91–92), converting a random coil into an α-helix. Thr390Ala induced no detectable conformational changes (Fig. 6). Owing to the absence of N-terminal residues (prior to position 37) in all nine high-homology models generated by SWISS-MODEL, the structural impact of Ala5Thr could not be assessed. Notably, Thr390Ala Markedly reduced hydrogen-bonding potential at residue 390, decreasing the number of hydrogen bonds with residue 382 from four to two And abolishing the two bonds with residue 387 (Fig. 7).

**Fig. 8:**
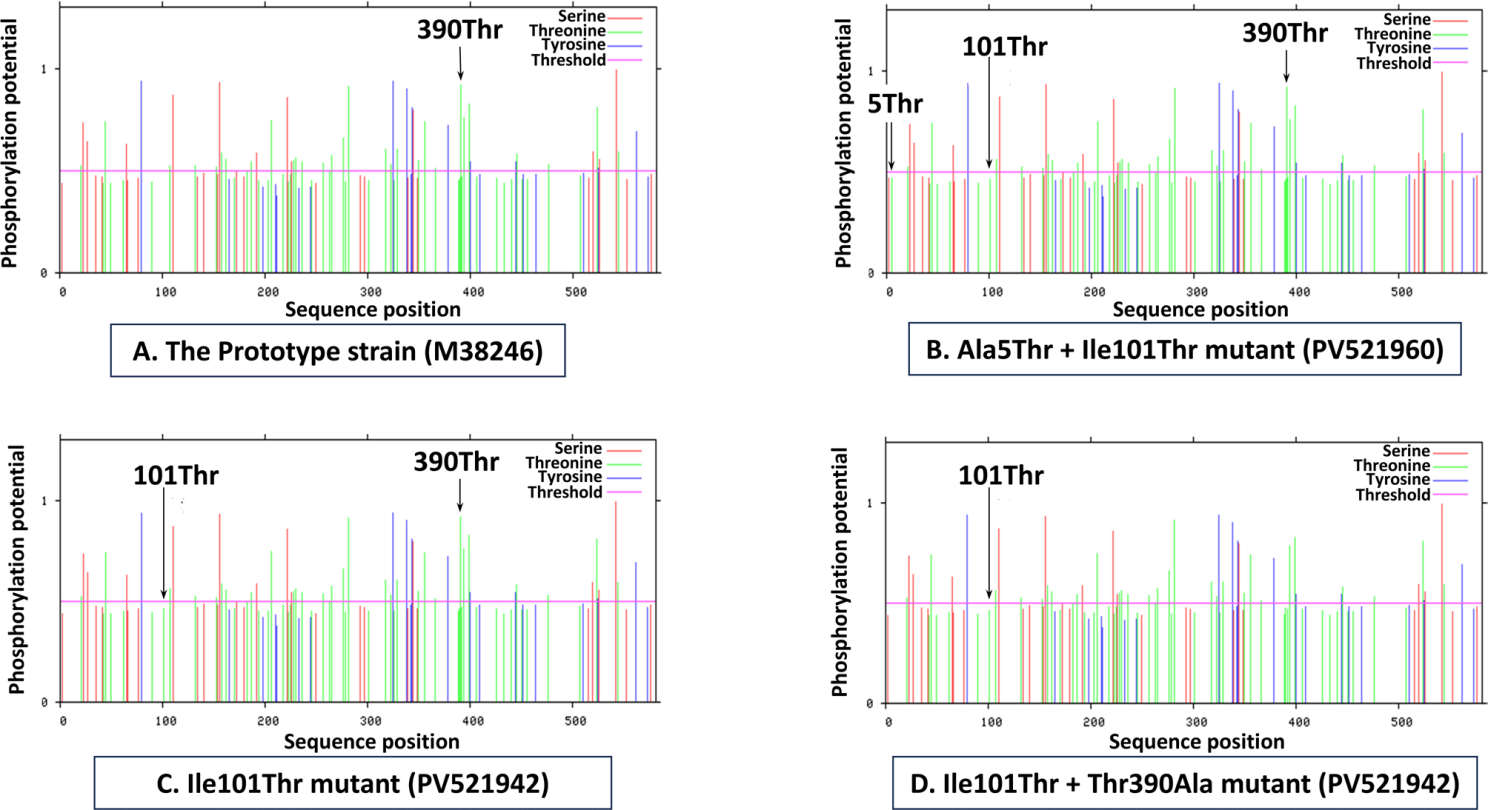
In silico prediction of phosphorylation potential at all three VP2 residues mutated in Egyptian FPV strains (positions 5, 101, And 390), based on the presence of threonine either as the original residue or introduced by mutation. Only residue 390 was predicted to be a potential phosphorylation site; thus, the Thr390Ala mutation abolished this phosphorylation potential (**D**)

Given that Ala5Thr, Ile101Thr, and Thr390Ala mutations involve threonine, a residue susceptible to phosphorylation, we assessed phosphorylation sites in mutant versus wild-type VP2. Only residue 390 was predicted as a key phosphorylation site. The Thr390Ala mutation abolished its phosphorylation potential (Fig. 8).

### Spatiotemporal distribution of the Thr390Ala mutant

**Fig. 9:**
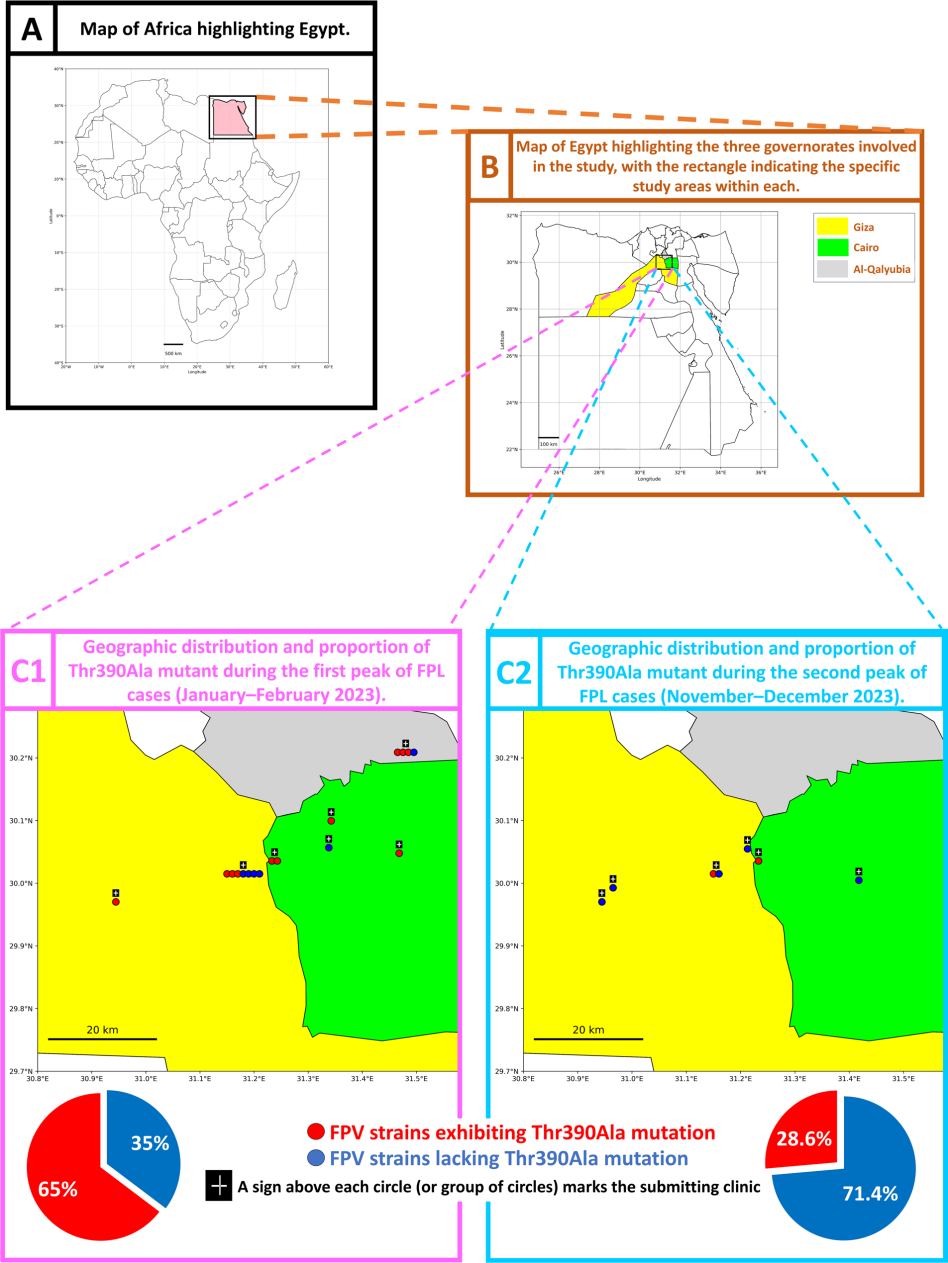
Spatial and temporal distribution of Egyptian FPV strains exhibiting the unique Thr390Ala mutation And other strains lacking this mutation during two peaks of FPL cases in 2023. As shown in panels C1/C2, the proportion of Thr390Ala mutants during the first peak was high (11/17, 65%), with a sharp decline during the second peak (2/7, 28.6%). Pie charts are added below each map in panel C1/C2 to visualize the proportion of mutant versus non-mutant strains during each peak

During the first peak of FPL cases (January–February 2023), FPV strains carrying the unique Thr390Ala mutation predominated, accounting for 65% of cases (11/17). However, its prevalence declined sharply during the second peak (November–December 2023), representing only 28.6% of cases (2/7). Figure 9 shows the spatial distribution of FPV strains with and without the Thr390Ala mutation across three Egyptian governorates during both peaks.

### Association between the Thr390Ala mutation and disease outcome

To assess whether the Thr390Ala mutation was associated with disease severity, we compared the clinical outcome (recovery vs. death/euthanasia) between FPV-positive cats infected with mutant strains (*n* = 13) and those infected with non-mutant strains (*n* = 11). The recovery rate was 45.4% (5/11) in the non-mutant group And 46.2% (6/13) in the mutant group. Statistical analysis using Fisher’s exact test showed no significant association between the presence of the Thr390Ala mutation and disease outcome (*p* = 1.00).

## Discussion

Depending on the age of the infected cat, FPV causes two distinct clinical syndromes: FPL and CA [15]. FPL should be the primary differential diagnosis in cats presenting with acute-onset gastroenteritis, as early detection and isolation are critical for controlling this highly contagious and often fatal disease [14]. A definitive diagnosis in non-vaccinated cats can typically be achieved shortly after admission using FPV or CPV Ag PoC kits [17]– [18]. In the current study, all cats admitted with acute gastroenteritis (*n* = 22) were ≤ six-month-old, non-vaccinated kittens, and all tested positive using FPV Ag PoC kits, confirming a diagnosis of FPL and reflecting the well-known FPV preference to infect young, non- or improperly vaccinated kittens [23–25].

FPL-affected kittens usually show fever, lethargy, anorexia, vomiting, diarrhea, and dehydration, a nonspecific clinical presentation overlapping with other causes of acute, non-hemorrhagic gastroenteritis, though symptoms are typically more severe [26]. Most affected cats present with a variable combination of these signs at admission, with vomiting generally more prominent than diarrhea, which often appears later in the course of illness and is rarely hemorrhagic, in contrast to canine parvoviral enteritis [14, 23, 26, 27]– [28]. Consistent with prior reports, the cats in this study did not exhibit the full spectrum of clinical signs at admission, displayed vomiting more consistently than diarrhea (100% vs. 68%), and rarely developed hemorrhagic diarrhea (2/22, 9%). Hypothermia and lateral recumbency, clinical indicators of a terminal disease stage, were observed in only two kittens (2/22, 9%), both of which had a fatal outcome. Hypothermia has been identified as a poor prognostic indicator of FPL [26]. Despite prompt hospitalization and supportive care, the survival rate in this cohort was low (12/22, 54.5%), aligning with previous studies, which reported survival rates ranging from 20 to 55% [15]. This reflects the severe and often catastrophic nature of FPL in cats, causing high mortality due to septic and hypovolemic shock [14].

CA is another clinical manifestation of FPV in cats, occurring when FPV infects the cerebellum, specifically the external germinal cell layer, during the perinatal period, i.e., during the last three weeks of gestation or the first two weeks after birth, when these cells are still mitotically active [15]. This leads to cerebellar hypoplasia, a developmental defect in the brain region responsible for balance and coordination [29]. In the current study, two pairs of littermate kittens from separate litters presented with signs of CA. The case history of one litter suggested prenatal infection, as the dam had been exposed to a cat with clinical FPL during late pregnancy. For the other litter, infection could have occurred pre- or postnatally, possibly due to ongoing exposure of the strictly indoor dam and kittens to an unvaccinated, adult, intact cat with outdoor access.

Epidemiological and clinical evidence from prior reports on FPV-associated CA indicate that the number of affected kittens per litter varies, and disease severity may range widely among littermates [30]. Although affected kittens are typically not in pain and often learn to adapt to their neurological impairment, severe cases may be euthanized due to poor quality of life [29]. Consistent with this, neither litter was entirely affected in the current study, with only two out of five kittens showing CA. Moreover, in both litters, one kitten displayed more pronounced neurological signs compared to its littermate, necessitating euthanasia.

Despite challenges in reaching a definitive antemortem diagnosis of FPV-associated cerebellar hypoplasia, FPV remains the most common cause of this recognizable congenital disorder [30]. In the present study, PCR was selected over antigen detection to confirm FPV-associated cerebellar atrophy in both kittens post-mortem, as the virus is known to persist in a latent form within cerebellar cortical cells [19]. This latent infection can be consistently detected in cerebellar tissue, with PCR Yielding positive results in 100% of examined samples in previous studies [19, 29].

Among the 1,024 FPV sequences in the original dataset, only five (0.5%) were identified as recombinant strains. This low frequency supports the notion that, unlike RNA viruses, e.g., influenza virus and coronavirus, recombination is not considered a central evolutionary mechanism in FPV. Notably, a recent study reported that three of these five recombinant strains (accession no. MH559110, MN400978, and MN400980) clustered within a distinct lineage, designated FPV-G3H, which appeared as an outlier to the main clades in the phylogenetic tree, providing external validation of their recombinant nature. The remaining two strains were not included in that study’s analysis [11].

The presence of one or more CPV-2-specific amino acids at key discriminating sites in 27 sequences from the original dataset suggests that some FPV strains may undergo convergent evolution or evolve due to recombination events between FPV and CPV-2, aimed at adapting to dogs or other animal species [24, 31–33]. Alternatively, they could represent misclassified CPV-2 strains, particularly in the case of the first seven strains Listed in Supplementary Table 3.

The dN/dS ratio calculated in this study was < 1, indicating that synonymous mutations outnumbered nonsynonymous ones, consistent with previous findings [7, 21, 24, 34]– [35]. This pattern suggests that the evolution of FPV is predominantly shaped by purifying (negative) selection, whereby nonsynonymous mutations are selectively removed to preserve the structural integrity and functional stability of the VP2 protein. Consequently, most observed mutations likely arise from random genetic drift rather than adaptive evolution. However, certain amino acid positions may evolve under positive selection pressure, as seen in the current or previous studies [36].

The Egyptian sequences generated in this study exhibited three nonsynonymous mutations relative to the prototype strain FPV-b. The first was An alanine-to-threonine substitution at position 5 (Ala5Thr) near the N-terminus of the VP2 protein. Epitope prediction indicated that residue 5 Lies within An Antigenic region spanning amino acids 5–33. The Ala5Thr substitution slightly increased the predicted epitope score (from 0.1578 to 0.1628), suggesting a potential enhancement in immune recognition. This may explain the limited circulation of this variant in our study population, where it was detected in only one Egyptian sequence (1/24, 4.1%).

Notably, residue 5 is part of a Linear epitope within the first 21 amino acids of the closely related CPV-2 VP2 protein, which was used to develop an early peptide-based CPV vaccine [37]– [38]. While experimental confirmation in FPV is lacking, this supports a potential immunological relevance.

Consistent with this, the Ala5Thr mutation was rare among global sequences (18/967, 1.8%), with most occurrences (16/18, 84.2%) reported in a large-scale study from Australia Analyzing 94 full-length VP2 sequences collected during FPL outbreaks between 2014 And 2018, primarily in shelter-housed cats [39].

Given that the Ala5Thr variant was observed predominantly in an Australian cluster that was not interpreted in the original study [39], we independently re-analyzed their metadata to investigate potential drivers of its emergence And apparent geographic restriction. These 16 sequences were confined to localized outbreaks in specific Victorian cities, Mildura And Geelong, during 2015–2016 (Supplementary Table 5). Notably, the mutation was absent from sequences obtained in Melbourne in 2017 and from New South Wales throughout the study period. Shelter vaccination practices may partly explain this distribution. In Mildura, none of the three studied shelters vaccinated cats, whereas in Melbourne, two of three shelters implemented vaccination on admission using either modified-live or inactivated vaccines. This contrast suggests that insufficient vaccination may have allowed brief circulation of this immunogenic variant in under-immunized regions, while more rigorous immunization in other areas likely impeded its spread. These observations further support the hypothesis that the Ala5Thr substitution increases immune detectability and reduces viral fitness.

By contrast, a different substitution at this position in CPV-2, Ala5Gly, is dominant in Asiatic-origin CPV-2c strains And is consistently observed alongside mutations at positions 267, 324, And 370 [40]– [41]. This mutation profile might have been associated with enhanced epidemic fitness and potentially increased virulence [41]. However, this remains a speculative hypothesis, as the precise functional role of the Ala5Gly mutation has not yet been elucidated [42].

The second nonsynonymous mutation affected the 101 st amino acid residue of the VP2, resulting in an isoleucine-to-threonine substitution (Ile101Thr). In the current study, this residue was identified as being under positive selection, suggesting that the Ile101Thr mutation May confer a fitness advantage to the virus. Supporting this hypothesis, this mutation was first reported in two American FPV strains isolated in 1964 (accession no. EU659112 and U22189) and has since rapidly spread and become predominant worldwide. In our dataset, the Ile101Thr mutation was observed in 100% of Egyptian sequences (24/24) And 95.6% of reference strains (925/967). The remaining 42 reference strains, either retaining isoleucine or showing alternative substitutions at this position (Supplementary Table 6), were genetically related to the prototype strain, all clustering within the FPV-G2 group in the ML tree.

In this study, in silico analyses revealed that the Ile101Thr mutation reduced the predicted epitope score at residues 91–93 And altered the local secondary structure at residues 91–92, transitioning from a random coil to an α-helix. This suggests that Ile101Thr modulates antigenicity in this loop1 region through structural rearrangement. Notably, residues 91–93 Lie within a prominent capsid loop, with position 93 being a key determinant of receptor binding, host range, and antigenicity [43]. In a previous study, threonine at position 101 has been shown to enable a polar contact with Asp99, altering the outer layer of the central areas responsible for receptor binding [44]. This likely enhances the interaction between VP2 And the transferrin receptor. Collectively, the potential for increased receptor affinity And reduced immune recognition at residue 93 may have contributed to the rapid global emergence and dominance of the Ile101Thr variant.

The third nonsynonymous mutation identified was particularly striking: a substitution of the highly conserved threonine at residue 390 of the VP2 protein with alanine (Thr390Ala), a change absent from all 967 global FPV reference sequences examined. Residue 390 lies within the GH loop (aa 267–498) of VP2, a large, surface-exposed, and immunologically active region known for its variability among parvoviruses [4, 21]. In silico 3D modeling revealed that while the overall VP2 protein structure remained intact, the Thr390Ala mutation caused localized alterations, specifically the loss of several hydrogen bonds and disruption of a predicted phosphorylation site, notably by replacing the hydroxyl group of threonine (capable of forming key hydrogen-bond networks) with a nonpolar methyl group in alanine, potentially destabilizing the GH loop. Similar functional disruptions have been reported in related parvoviruses; for example, mutations at C-terminal phosphorylation sites in the canine parvovirus NS1 protein have been shown to reduce viral replication and pathogenicity [45].

Despite this, the mutant virus demonstrated sustained transmissibility And pathogenicity, dominating the first FPL peak of 2023 (11/17 sequences; 65%) and exhibiting clinical outcomes comparable to those of wild-type strains, suggesting compensatory advantages. Epitope prediction analyses further indicated that the mutation resides within a predicted antigenic hotspot, slightly reducing epitope score and suggesting a plausible mechanism of immune evasion that could compensate for structural instability. Previous studies on capsid–antibody complex structures may provide a mechanistic explanation for how the Thr390Ala mutation May contribute to immune escape, either directly, as residue 390 is among the contact residues of the neutralizing monoclonal antibody Fab-16, or indirectly, by disrupting all hydrogen bonds with residue 387, which itself a contact site for two other potent neutralizing antibodies, Fab-E and Fab-F [46]– [47]. While all cats in this study were unvaccinated, some may have retained maternally derived antibodies, and cats in the broader population may have vaccine-induced immunity, potentially exerting immune pressure favoring escape variants.

All Egyptian sequences bearing this mutation clustered into a distinct, previously unrecognized subclade in the phylogenetic tree. Along with its absence from the worldwide reference sequences, including the only previous Egyptian sequences (accession no. OM937916 and OM638043, collected in 2019 And 2021, resepectively), this phylogenetic pattern underscores both the novelty and recent emergence of the mutant, Likely arising from a shared recent evolutionary origin followed by rapid local spread during the first 2023 peak. While FPL cases in Egypt typically reach peak activity during the winter season (December to February), field observations, personal communications with veterinarians (M. Safwat), And social media reports consistently described An unusual surge in FPL cases between November 2022 And February 2023 compared to prior seasons, coinciding with the emergence and rapid rise of the Thr390Ala-bearing mutant, which accounted for 65% of sequenced cases during the first 2023 peak. This pattern suggests a selective sweep driven by the emerging mutant during this period.

However, the apparent decline in its frequency during the second 2023 peak (2/7 sequences, 25%) highlights a classic evolutionary trade-off: while the mutation may have conferred temporary immune escape, it likely compromised capsid stability or replication efficiency, ultimately reducing long-term viral fitness. This might allow other variants to regain dominance. This interpretation is further supported by the lack of positive selection at this site, even in MEME, which is designed to detect episodically advantageous mutations arising during localized outbreaks.

The Thr390Ala substitution identified in this study offers a rare opportunity to observe viral evolution in real time, capturing the emergence, spread, and decline of a potentially immune-driven variant. It underscores that even viruses with a reputation for genomic stability, like FPV, are not entirely static and can undergo short-lived but epidemiologically significant shifts. From a disease control perspective, it reinforces the value of proactive genetic surveillance and structural modeling, especially in under-sampled regions. Such approaches can reveal transient mutants that might otherwise go unnoticed yet still provide critical lessons.

While our supposed hypothese regarding the trajectory of recent emergence, transient dominance, and subsequent decline, is based on the novelty of the Thr390Ala mutant, along with its distinct epidemiological pattern, phylogenetic placement, and in silico analysis, the small sample size and the unvaccinated status of all animals prompt larger-scale field investigations supported with in vivo or in vitro experimental analysis to validate and expand upon these findings.

To contextualize the emergence of the Thr390Ala mutant, we examined the global FPV VP2 sequence dataset and found that no other notable lineage emergence has been reported in recent decades, except for the Ala91Ser variant, particularly in China (Supplementary Fig. 1). These two mutations, Thr390Ala and Ala91Ser, therefore represent the only known examples of distinct FPV variant emergence with both phylogenetic distinction and epidemiological significance. However, their evolutionary trajectories diverged markedly. Thr390Ala recently emerged in Egyptian cats And exhibited a transient rise And rapid decline, briefly dominating the first FPL peak of 2023 in Egypt, whereas Ala91Ser, first detected in China in 2005, recently rapidly established itself as the dominant strain in China and remains prevalent over the few previous years [5, 11, 33, 36, 48]– [49]. Structural modeling offers a potential mechanistic explanation: Ala91Ser induces substantial remodeling of the local VP2 conformation, stabilizing a random coil at residue 93, which may enhance receptor-binding avidity [5, 11]. In contrast, Thr390Ala likely causes localized destabilization of the GH loop. While Thr390Ala was not associated with altered pathogenicity, Ala91Ser has been linked to increased infectivity and disease severity in cats [49]. Finally, residue 91 has been previously shown to be under positive selection [36], whereas residue 390 has not. This comparison highlights how different single amino acid substitutions can dramatically influence viral evolution in distinct ways, one potentially leading to a short-term regional sweep (Thr390Ala), and the other enabling long-term regional dominance (Ala91Ser).

We further examined whether any of the identified VP2 mutations were associated with distinct clinical syndromes, particularly cerebellar ataxia. No distinct substitutions uniquely associated with cerebellar involvement, suggesting that the VP2 protein alone may not be responsible for the development of cerebellar ataxia in FPV-infected kittens. However, it remains plausible that genetic determinants of neurotropism and cerebellar pathology reside in other regions of the viral genome, particularly within the non-structural proteins NS1 and NS2. To date, no published studies have reported complete genome sequences of FPV strains isolated from cases of cerebellar ataxia in cats, despite the clinical significance of this manifestation [19, 29]. A study by Garigliany et al. (2013) identified a unique L582S substitution in the NS1 protein of FPV strains from four cats with strong viral antigen staining in cerebral neurons, a mutation not found in intestinal strains from cats lacking such staining [50]. Although these findings pertained to cerebral rather than cerebellar tissue, they underscore the potential role of non-structural proteins in modulating tissue tropism. Further genomic investigations focusing on FPV strains derived from cerebellar tissue are therefore warranted to elucidate possible molecular determinants of this neurologic syndrome.

FPV sequences in the final dataset were isolated from various Animal species, including domestic cats And dogs, and 22 different wild animal species, indicating the broad host range of this virus. For several decades, it has been speculated that FPV is not pathogenic for domestic dogs since it cannot replicate in the small intestine. However, most canine-origin reference FPV strains in the final dataset were collected from fecal samples of dogs showing severe signs of gastroenteritis (Supplementary Table 7). None of these canine FPV strains were collected before 2017, indicating that FPV might have recently acquired novel biological properties enabling it to cause clinical disease in dogs indistinguishable from canine parvoviral enteritis [36, 44, 51].

FPV strains in the final dataset were classified into three groups in the ML-based phylogenetic tree, consistent with previous studies [6–12]. However, our analysis did not identify group-specific nucleotide markers or strong bootstrap support for these groupings. To explore whether the weak support resulted from the inclusion of a large number of highly similar sequences, we generated a reduced tree using a subset of more genetically distinct representatives. Despite this adjustment, bootstrap support remained low (Fig. 4). The low bootstrap values in VP2-based FPV phylogenies are not unique to our dataset and have been consistently reported in several previous studies [8–11, 49]. These findings suggest that the observed grouping is based on topology rather than robust phylogenetic signals.

Nevertheless, we retained this grouping scheme to assess its potential strengths and limitations. We found this grouping could serve as a meaningful heuristic framework to explore emerging spatiotemporal, genetic, and biological trends. Spatiotemporally, FPV-G3 remains the most prevalent group globally, while FPV-G2 is the least represented; FPV-G1, although intermediate in size, has shown a recent and notable expansion, particularly in China, suggesting localized emergence [5]. Genetically, FPV-G1 strains were overwhelmingly associated with the VP2-Ala91Ser mutation (293/311, 94.2%), a trend not observed in other groups (e.g., only 4/498 in G3), suggesting a possible lineage-associated trend [5, 11, 33, 36, 48]– [49]. Biologically, FPV-G1 strains have been associated with increased disease severity in cats compared to G2 strains in experimental infection models [49], suggesting possible clinical relevance. Additionally, all currently available commercial FPV vaccines cluster within FPV-G2, while most circulating field strains belong to G1 and G3, raising concerns about a potential mismatch between vaccine and field strains [9]– [10, 12, 49]. Collectively, these consistent and multi-dimensional trends support the utility of this grouping scheme as a practical tool for tracking FPV evolution and informing epidemiological, immunological, and pathogenicity-focused investigations, despite its limited phylogenetic resolution. Nonetheless, future studies are warranted to refine FPV phylogeny using broader genomic regions and alternative analytical approaches such as Bayesian inference.

Egyptian strains clustered into two distinct subclades, 1 And 2. The unique nonsynonymous mutation A1168G/Thr390Ala characterized the emergence of the novel subclade 1, while the synonymous mutation C135T was the Major determinant of subclade 2. Among the 51 strains carrying the C135T mutation in the final dataset, those exhibiting the nonsynonymous mutations A927G and/or A1236G consistently clustered within subclade 2, except for one Egyptian sequence in this study (accession no. PV521947) (Supplementary Table 8). Instead, this sequence clustered within subclade 1, likely due to its concurrent possession of the nonsynonymous mutation A1168G/Thr390Ala, suggesting that this mutation may exert a stronger phylogenetic signal, contributing more substantially to this strain’s evolutionary placement. Notably, the predominance of C135T among strains in Egypt and the UAE [39] suggests it may serve as a potential genetic marker for FPV field strains in the Middle East (Supplementary Fig. 2). However, additional studies with larger sample sizes from both countries and other Middle Eastern countries are needed to validate this hypothesis and establish broader regional patterns.

The relatively small sample size and the fact that all studied cats were unvaccinated represent key limitations of this study. Nevertheless, it provides the first and largest collection of full-length VP2 sequences from Egypt, an understudied and previously undersampled region. Future studies with larger cohorts, including vaccinated animals, are required to validate the observed mutation patterns and their potential associations with viral evolution.

The second limitation is that, while FPV is the most likely and should be the primary suspected cause of severe gastroenteritis in cats due to its highly contagious and devastating nature, we acknowledge that other enteric pathogens (e.g., feline coronavirus, intestinal parasites, and enteric bacteria) were not excluded in this study. Such co-infections may contribute to variability in clinical presentation or influence survival outcomes.

## Conclusions

The current study addresses the limited data on FPV genetic diversity and evolution in Egyptian cats by providing the largest number of full-length *VP2* sequences from Egypt to date, further enriching the global FPV sequence repository And enhancing the ability to monitor viral evolutionary trends. In-depth genetic Analysis of Egyptian sequences, alongside a worldwide dataset of 967 reference sequences, enabled us to identify the unique mutation Thr390Ala, which was phylogenetically distinct enough to define a novel subclade. This mutant likely represents a classic evolutionary trade-off: it emerged and predominated, possibly due to immune evasion advantages, but declined later, likely due to structural constraints and replacement by more competitive variants. Despite FPV typically exhibiting strong purifying selection (dS/dN ratio = 0.121), this study’s findings indicate that FPV is not entirely genetically static. This investigation moves beyond sequence documentation; it provides a valuable case study in viral adaptation, immune interaction, and evolutionary constraints, enriching our capacity for informed surveillance, outbreak response, and future vaccine design.

## Supplementary Information

Supplementary Material 1.

Supplementary Material 2.

Supplementary Material 3.

Supplementary Material 4.

Supplementary Material 5.

Supplementary Material 6.

Supplementary Material 7.

Supplementary Material 8.

Supplementary Material 9.

Supplementary Material 10.

Supplementary Material 11.

Supplementary Material 12.

Supplementary Material 13.

Supplementary Material 14.

Supplementary Material 15.

## Data Availability

DNA sequences generated in this study were submitted to GenBank (NCBI) following accession: PV521942-PV521965.
