# Supplementary material for: Emergence, surge, and fading of the novel feline parvovirus Thr390Ala mutant in Egyptian cats during 2023: insights from a comprehensive full-length VP2 genetic analysis
Source: BMC Vet Res. 2025 Oct 3;21:570. doi: 10.1186/s12917-025-05004-3 (PMC12492670; doi:10.1186/s12917-025-05004-3)
Supplement: Supplementary file 5 — Supplementary Material 5. [file 12917_2025_5004_MOESM5_ESM.docx]

**Supplementary Figure 1. The frequency of FPV strains exhibiting the synonymous mutation C135T among all sequences characterized in each country. C135T was detected only in FPV strains from eight countries.**
