# Supplementary material for: Emergence, surge, and fading of the novel feline parvovirus Thr390Ala mutant in Egyptian cats during 2023: insights from a comprehensive full-length VP2 genetic analysis
Source: BMC Vet Res. 2025 Oct 3;21:570. doi: 10.1186/s12917-025-05004-3 (PMC12492670; doi:10.1186/s12917-025-05004-3)
Supplement: Supplementary file 6 — Supplementary Material 6. [file 12917_2025_5004_MOESM6_ESM.docx]

**Supplementary Table 5**

**Rare feline parvovirus reference strains with threonine (Thr) at residue 5 of the VP2 protein**

| Strain name | Country | Date | Host | FPV group | GenBank acc. no. |
| --- | --- | --- | --- | --- | --- |
| FPV_C7 | Australia | 2015 | *Felis catus* | G2 | MK570637 |
| FPV_C5 | Australia | 2015 | *Felis catus* | G2 | MK570638 |
| FPV_C4 | Australia | 2015 | *Felis catus* | G2 | MK570639 |
| FPV_C3 | Australia | 2015 | *Felis catus* | G2 | MK570640 |
| FPV_C2 | Australia | 2015 | *Felis catus* | G2 | MK570641 |
| FPV_C1 | Australia | 2015 | *Felis catus* | G2 | MK570642 |
| FPV_LK | Australia | 2015 | *Felis catus* | G2 | MK570643 |
| FPV_31 | Australia | 2015 | *Felis catus* | G2 | MK570646 |
| FPV_33 | Australia | 2015 | *Felis catus* | G2 | MK570647 |
| FPV_75 | Australia | 2016 | *Felis catus* | G2 | MK570648 |
| FPV_76 | Australia | 2016 | *Felis catus* | G2 | MK570649 |
| FPV_77 | Australia | 2016 | *Felis catus* | G2 | MK570650 |
| FPV_78 | Australia | 2016 | *Felis catus* | G2 | MK570651 |
| FPV_79 | Australia | 2016 | *Felis catus* | G2 | MK570652 |
| FPV_80 | Australia | 2016 | *Felis catus* | G2 | MK570653 |
| FPV_185 | Australia | 2016 | *Felis catus* | G2 | MK570749 |
| FPV_5371/20 | Australia | 2019 | *Canis lupus familiaris* | G2 | MZ362883 |
| FPV/979 | USA | 2013 | *Felis catus* | G2 | KJ813893 |

These strains exhibit a **Thr (T)** at VP2 position 5, where **Ala (A)** is otherwise highly conserved in FPV.
