# Supplementary material for: Emergence, surge, and fading of the novel feline parvovirus Thr390Ala mutant in Egyptian cats during 2023: insights from a comprehensive full-length VP2 genetic analysis
Source: BMC Vet Res. 2025 Oct 3;21:570. doi: 10.1186/s12917-025-05004-3 (PMC12492670; doi:10.1186/s12917-025-05004-3)
Supplement: Supplementary file 7 — Supplementary Material 7. [file 12917_2025_5004_MOESM7_ESM.docx]

**Supplementary Table 8**

**List of all Egyptian and reference sequences in the final dataset exhibiting the nonsynonymous mutation C135T and their clustering into subclades 2 based on the concurrent presence of the nonsynonymous mutations A927G and or A1236G**

| Strain name | Country | Date | Host | FPV group |  | C135T | A927G | A1236G | Subclade |
| --- | --- | --- | --- | --- | --- | --- | --- | --- | --- |
| GE1001 this study | Egypt | 2023 | *Felis catus* | G3 | PV521942 | + | + | - | Subclade 2 |
| GE1007 this study | Egypt | 2023 | *Felis catus* | G3 | PV521948 | + | + | + | Subclade 2 |
| GE1008 this study | Egypt | 2023 | *Felis catus* | G3 | PV521949 | + | + | - | Subclade 2 |
| GE1012 this study | Egypt | 2023 | *Felis catus* | G3 | PV521953 | + | + | + | Subclade 2 |
| GE1013 this study | Egypt | 2023 | *Felis catus* | G3 | PV521954 | + | + | + | Subclade 2 |
| GE1014 this study | Egypt | 2023 | *Felis catus* | G3 | PV521955 | + | + | - | Subclade 2 |
| GE1019 this study | Egypt | 2023 | *Felis catus* | G3 | PV521960 | + | + | - | Subclade 2 |
| GE1020 this study | Egypt | 2023 | *Felis catus* | G3 | PV521961 | + | + | - | Subclade 2 |
| GE1021 this study | Egypt | 2023 | *Felis catus* | G3 | PV521962 | + | + | + | Subclade 2 |
| GE1022 this study | Egypt | 2023 | *Felis catus* | G3 | PV521963 | + | + | - | Subclade 2 |
| CA1023 this study | Egypt | 2023 | *Felis catus* | G3 | PV521964 | + | + | + | Subclade 2 |
| FPV_278 | Australia | 2018 | *Felis catus* | G3 | MK570675 | + | + | - | Subclade 2 |
| SDYT22 | China | 2022 | *Felis catus* | G3 | OQ535507 | + | - | + | Subclade 2 |
| SDYT3 | China | 2022 | *Felis catus* | G3 | OR211671 | + | - | + | Subclade 2 |
| 39-566 | Egypt | 2019 | *Felis catus* | G3 | OM937916 | + | + | - | Subclade 2 |
| 139-188 | Egypt | 2021 | *Canis lupus familiaris* | G3 | OM638043 | + | + | + | Subclade 2 |
| BFPV | Finland | 1983 | *Vulpes lagopus* | G3 | MN451652 | + | - | + | Subclade 2 |
| 18R217C | Thailand | 2018 | *Felis catus* | G3 | MN127779 | + | + | + | Subclade 2 |
| 19R81C | Thailand | 2019 | *Felis catus* | G3 | MN127780 | + | + | - | Subclade 2 |
| TRC-B88 | Thailand | 2020 | *Felis catus* | G3 | MW589472 | + | + | + | Subclade 2 |
| FPV/UAE/2017 | UAE | 2017 | *Felis catus* | G3 | MN603975 | + | + | + | Subclade 2 |
| FPV/UAE/2017 | UAE | 2017 | *Felis catus* | G3 | MK570716 | + | + | + | Subclade 2 |
| FPV/UAE/2017 | UAE | 2017 | *Felis catus* | G3 | MK570717 | + | + | + | Subclade 2 |
| FPV/UAE/2017 | UAE | 2017 | *Felis catus* | G3 | MK570718 | + | + | + | Subclade 2 |
| FPV/UAE/2017 | UAE | 2017 | *Felis catus* | G3 | MK570719 | + | + | + | Subclade 2 |
| FPV/UAE/2017 | UAE | 2017 | *Felis catus* | G3 | MK570720 | + | + | + | Subclade 2 |
| FPV/UAE/2017 | UAE | 2017 | *Felis catus* | G3 | MK570721 | + | + | + | Subclade 2 |
| FPV/UAE/2017 | UAE | 2017 | *Felis catus* | G3 | MK570722 | + | + | + | Subclade 2 |
| FPV/UAE/2017 | UAE | 2017 | *Felis catus* | G3 | MK570723 | + | + | + | Subclade 2 |
| FPV/UAE/2017 | UAE | 2017 | *Felis catus* | G3 | MK570724 | + | + | + | Subclade 2 |
| FPV/UAE/2017 | UAE | 2017 | *Felis catus* | G3 | MK570725 | + | + | + | Subclade 2 |
| GE1006 this study | Egypt | 2023 | *Felis catus* | G3 | PV521947 | + | + | + | Subclade 1 (A1236G/Thr390Ala mutation) |
| GE1002 this study | Egypt | 2023 | *Felis catus* | G3 | PV521943 | + | - | - | Subclade 1 (A1236G/Thr390Ala mutation) |
| GE1003 this study | Egypt | 2023 | *Felis catus* | G3 | PV521944 | + | - | - | Subclade 1 (A1236G/Thr390Ala mutation) |
| GE1004 this study | Egypt | 2023 | *Felis catus* | G3 | PV521945 | + | - | - | Subclade 1 (A1236G/Thr390Ala mutation) |
| GE1005 this study | Egypt | 2023 | *Felis catus* | G3 | PV521946 | + | - | - | Subclade 1 (A1236G/Thr390Ala mutation) |
| GE1010 this study | Egypt | 2023 | *Felis catus* | G3 | PV521951 | + | - | - | Subclade 1 (A1236G/Thr390Ala mutation) |
| GE1011 this study | Egypt | 2023 | *Felis catus* | G3 | PV521952 | + | - | - | Subclade 1 (A1236G/Thr390Ala mutation) |
| GE1015 this study | Egypt | 2023 | *Felis catus* | G3 | PV521956 | + | - | - | Subclade 1 (A1236G/Thr390Ala mutation) |
| GE1016 this study | Egypt | 2023 | *Felis catus* | G3 | PV521957 | + | - | - | Subclade 1 (A1236G/Thr390Ala mutation) |
| GE1017 this study | Egypt | 2023 | *Felis catus* | G3 | PV521958 | + | - | - | Subclade 1 (A1236G/Thr390Ala mutation) |
| GE1018 this study | Egypt | 2023 | *Felis catus* | G3 | PV521959 | + | - | - | Subclade 1 (A1236G/Thr390Ala mutation) |
| CA1024 this study | Egypt | 2023 | *Felis catus* | G3 | PV521965 | + | - | - | Subclade 1 (A1236G/Thr390Ala mutation) |
| ZJFPV7 | China | 2017 | *Felis catus* | G3 | MW495833 | + | - | - | Not specified |
| BJ020 | China | 2019 | *Felis catus* | G3 | MT270583 | + | - | - | Not specified |
| BJ016 | China | 2019 | *Felis catus* | G3 | MT270584 | + | - | - | Not specified |
| BJ006 | China | 2019 | *Felis catus* | G3 | MT270585 | + | - | - | Not specified |
| HNZZ2 | China | 2020 | *Panthera leo* | G3 | MZ005633 | + | - | - | Not specified |
| YCYH | China | 2021 | *Felis catus* | G3 | OR227624 | + | - | - | Not specified |
| Yanji5 | China | 2021 | *Felis catus* | G3 | OM885375 | + | - | - | Not specified |
| 39897 | Portugal | 2006 | *Felis catus* | G3 | EU221280 | + | - | - | Not specified |
| 351 | Spain | 2013 | *Meles meles* | G3 | KP682520 | + | - | - | Not specified |
