# Supplementary material for: Emergence, surge, and fading of the novel feline parvovirus Thr390Ala mutant in Egyptian cats during 2023: insights from a comprehensive full-length VP2 genetic analysis
Source: BMC Vet Res. 2025 Oct 3;21:570. doi: 10.1186/s12917-025-05004-3 (PMC12492670; doi:10.1186/s12917-025-05004-3)
Supplement: Supplementary file 8 — Supplementary Material 8. [file 12917_2025_5004_MOESM8_ESM.docx]

**Supplementary Table 6**

**Rare reference FPV strains retaining the prototype residue isoleucine or exhibiting non-threonine substitutions at VP2 position 101**

| Strain name | Country | Date | Host | FPV group | GenBank acc. no. | Amino acid substitution |
| --- | --- | --- | --- | --- | --- | --- |
| 193/70 | Australia | 1970 | *Felis catus* | G2 | X55115 | Isoleucine |
| FPV | Australia | 2015 | *Felis catus* | G2 | MK570644 | Isoleucine |
| FPV | Australia | 2015 | *Felis catus* | G2 | MK570645 | Isoleucine |
| FPV_254 | Australia | 2017 | *Felis catus* | G2 | MK570709 | Methionine |
| FPV_154 | Australia | 2017 | *Felis catus* | G2 | MK570737 | Isoleucine |
| FPV_331 | Australia | 2018 | *Felis catus* | G2 | MK570676 | Isoleucine |
| FPV_250 | Australia | 2018 | *Felis catus* | G2 | MK570707 | Isoleucine |
| FPV_259 | Australia | 2018 | *Felis catus* | G2 | MK570714 | Isoleucine |
| FPV_260 | Australia | 2018 | *Felis catus* | G2 | MK570715 | Valine |
| BFPV | China | 2008 | *Felis catus* | G2 | GQ857595 | Isoleucine |
| FPV003 | China | 2021 | *Felis catus* | G2 | OQ398386 | Isoleucine |
| FPV008 | China | 2021 | *Felis catus* | G2 | OQ398387 | Isoleucine |
| FPV013 | China | 2021 | *Felis catus* | G2 | OQ398388 | Isoleucine |
| FPV014 | China | 2021 | *Felis catus* | G2 | OQ398389 | Isoleucine |
| FPV021 | China | 2021 | *Felis catus* | G2 | OQ398390 | Isoleucine |
| FPV026 | China | 2021 | *Felis catus* | G2 | OQ398391 | Isoleucine |
| FPV027 | China | 2021 | *Felis catus* | G2 | OQ398392 | Isoleucine |
| FPV028 | China | 2021 | *Felis catus* | G2 | OQ398393 | Isoleucine |
| FPV046 | China | 2021 | *Felis catus* | G2 | OQ398418 | Isoleucine |
| FPV048 | China | 2021 | *Felis catus* | G2 | OQ398419 | Isoleucine |
| FPV049 | China | 2021 | *Felis catus* | G2 | OQ398420 | Isoleucine |
| FPV050 | China | 2021 | *Felis catus* | G2 | OQ398421 | Isoleucine |
| TU8 | Japan | 1976 | *Felis catus* | G2 | AB000070 | Isoleucine |
| Fukagawa | Japan | 1993 | *Felis catus* | G2 | AB000054 | Isoleucine |
| C1 | Nigeria | 2014 | *Felis catus* | G2 | OP985508 | Isoleucine |
| C2 | Nigeria | 2014 | *Felis catus* | G2 | OP985509 | Isoleucine |
| C3 | Nigeria | 2014 | *Felis catus* | G2 | OP985510 | Isoleucine |
| C5 | Nigeria | 2014 | *Felis catus* | G2 | OP985511 | Isoleucine |
| C10 | Nigeria | 2014 | *Felis catus* | G2 | OP985512 | Isoleucine |
| C14 | Nigeria | 2014 | *Felis catus* | G2 | OP985513 | Isoleucine |
| C18 | Nigeria | 2014 | *Felis catus* | G2 | OP985514 | Isoleucine |
| C32 | Nigeria | 2014 | *Felis catus* | G2 | OP985515 | Isoleucine |
| C51 | Nigeria | 2014 | *Felis catus* | G2 | OP985517 | Isoleucine |
| C58 | Nigeria | 2014 | *Felis catus* | G2 | OP985518 | Isoleucine |
| C64 | Nigeria | 2014 | *Felis catus* | G2 | OP985519 | Isoleucine |
| C78 | Nigeria | 2014 | *Felis catus* | G2 | OP985521 | Isoleucine |
| 50/07-2 | UK | 2007 | *Felis catus* | G2 | EU498717 | Isoleucine |
| FPV-b | USA | 1967 | *Felis catus* | G2 | M38246 | Isoleucine |
| FPV-8b | USA | 1989 | *Felis catus* | G2 | EU659114 | Isoleucine |
| Purevax® | Vaccine | Vaccine | Vaccine | G2 | EU498680 | Isoleucine |
| Feligen® | Vaccine | Vaccine | Vaccine | G2 | ON605652 | Isoleucine |
| Phillips Rox | Vaccine | Vaccine | Vaccine | G2 | M24002 | Isoleucine |
