# Supplementary material for: Emergence, surge, and fading of the novel feline parvovirus Thr390Ala mutant in Egyptian cats during 2023: insights from a comprehensive full-length VP2 genetic analysis
Source: BMC Vet Res. 2025 Oct 3;21:570. doi: 10.1186/s12917-025-05004-3 (PMC12492670; doi:10.1186/s12917-025-05004-3)
Supplement: Supplementary file 9 — Supplementary Material 9. [file 12917_2025_5004_MOESM9_ESM.docx]

**Supplementary Table 3**

**Feline parvovirus-like strains**

| Strain name | Country/Year | Host | GenBank acc. no. | FPV-specific amino acids | | | | | |
| --- | --- | --- | --- | --- | --- | --- | --- | --- | --- |
|  |  |  |  | 80 (K) | 93 (K) | 103 (V) | 323 (D) | 564 (N) | 568 (A) |
| SMU-D14 | China/2018 | *Felis catus* | MZ442308 | R | N | A | N | S | G |
| SMU-D14 | China/2018 | *Felis catus* | MZ442310 | R | N | A | N | S | G |
| DL04 | China/2018 | *Felis catus* | ON646204 | R | N | A | N | S | G |
| Luoyang-13 | China/2020 | *Felis catus* | OR551220 | R | N | A | N | S | G |
| Zhengzhou-26 | China/2021 | *Felis catus* | OR551225 | R | N | A | N | S | G |
| Yanji2 | China/2021 | *Felis catus* | OM322821 | R | N | A | N | S | G |
| RACFPV1 | USA/2011 | *Procyon lotor* | MN451692 | R | N | A | N | S | G |
| LZ19 | China/2022 | *Felis catus* | OR783315 | R | N | A | D | N | A |
| C71 | Nigeria/2014 | *Felis catus* | OP985520 | R | N | A | D | N | A |
| Rac2.2 | USA/1978 | *Procyon lotor* | JN867595 | K | K | V | N | N | A |
| Rac1.2 | USA/1978 | *Procyon lotor* | JN867596 | K | K | V | N | N | A |
| V142 | Vietnam/1997 | *Felis catus* | AB054225 | K | K | V | N | N | A |
| C39 | Nigeria/2014 | *Felis catus* | OP985516 | K | K | V | N | N | A |
| Rac3 | USA/1978 | *Procyon lotor* | KM624023 | K | K | V | N | N | A |
| JL-04/17-03 | China/2017 | *Felis catus* | MF541126 | K | K | V | D | S | G |
| Jilin5 | China/2018 | *Felis catus* | MK266799 | K | K | V | D | S | G |
| FPV-SD/3 | China/2019 | *Felis catus* | OK384311 | K | K | V | D | S | G |
| FPV-SD/5 | China/2019 | *Felis catus* | OK384313 | K | K | V | D | S | G |
| FPV-SD/6 | China/2019 | *Felis catus* | OK384314 | K | K | V | D | S | G |
| BJ240 | China/2019 | *Felis catus* | MT270571 | K | K | V | D | S | G |
| HN2105 | China/2021 | *Felis catus* | OQ868567 | K | K | V | D | S | G |
| 1335/07 | Hungary/2007 | *Felis catus* | EU360959 | K | K | V | D | S | G |
| FPV-SD/2 | China/2019 | *Felis catus* | OK384310 | K | K | V | D | N | G |
| FPV-SD/4 | China/2019 | *Felis catus* | OK384312 | K | K | V | D | N | G |
| K50 | Korea/2008 | *Felis catus* | HQ184196 | Q | K | V | D | N | A |
| C92 | Nigeria/2014 | *Felis catus* | OP985522 | K | N | V | D | N | A |
| 389/07 | Hungary/2007 | *Paradoxurus hermaphroditus* | EU145593 | K | K | V | D | S | A |

**FPV-like strains exhibiting one or more CPV-2-specific amino acid residues at key discriminatory aa residues**. Substitutions were identified through multiple sequence alignment of the VP2 protein, and affected residues are **highlighted in yellow**.
