# Supplementary material for: Emergence, surge, and fading of the novel feline parvovirus Thr390Ala mutant in Egyptian cats during 2023: insights from a comprehensive full-length VP2 genetic analysis
Source: BMC Vet Res. 2025 Oct 3;21:570. doi: 10.1186/s12917-025-05004-3 (PMC12492670; doi:10.1186/s12917-025-05004-3)
Supplement: Supplementary file 10 — Supplementary Material 10. [file 12917_2025_5004_MOESM10_ESM.docx]

**Supplementary Table 2**

**Recombinant FPV strains identified by RDP v5 and excluded from the final dataset**

| Recombinant strainᵃ | | | Breakpoints  (start-end) | Major parent | Minor parent | Detection methods (*P* value)ᵇ | | | | |
| --- | --- | --- | --- | --- | --- | --- | --- | --- | --- | --- |
| Accession no. | Name | Country/year |  |  |  | RDP | MaxChi | Chimaera | SiScan | 3Seq |
| OQ266795 | ABT/MVC/01 | India/2018 | 15–200 | Unknown | MK425502 | – | + (7.6E-4) | + (2E-3) | + (3.1E-3) | + (6E-4) |
| MH559110 | TN/FPV/2018 | India/2018 | 15–200 | Unknown | MK425502 | – | + (7.6E-4) | + (2E-3) | + (3.1E-3) | + (6E-4) |
| MN400980 | Rara | Korea/2017 | 9–538 | HQ184204 | Unknown | + (2.1E-3) | + (6.4E-4) | + (9.3E-4) | + (1.4E-7) | – |
| MN400978 | Gigucheon | Korea/2017 | 1182–1226 | MW495838 | Unknown | + (9.4E-4) | + (6.4E-4) | + (9.3E-4) | + (4.7E-7) | – |
| MK425501 | CMU-F07 | Thailand/2018 | 39–457 | Unknown | MK425498 | – | + (3.3E-3) | + (5.2E-3) | + (7.9E-3) | + (3E-3) |

**ᵃ** Strains identified as recombinant by at least three methods with *P* < 0.01. GENECONV did not detect any of the five recombination events and is therefore not shown in the table.

ᵇ“+” indicates detection (*P*-value in parentheses); “–” indicates not detected by the corresponding method.
