# Supplementary material for: Emergence, surge, and fading of the novel feline parvovirus Thr390Ala mutant in Egyptian cats during 2023: insights from a comprehensive full-length VP2 genetic analysis
Source: BMC Vet Res. 2025 Oct 3;21:570. doi: 10.1186/s12917-025-05004-3 (PMC12492670; doi:10.1186/s12917-025-05004-3)
Supplement: Supplementary file 11 — Supplementary Material 11. [file 12917_2025_5004_MOESM11_ESM.docx]

**Supplementary Table 1**

| Primer name | Sequence 5′–3′ | Positionᵃ | Amplicon size | The number of amplification cycles/annealing temp | Reference |
| --- | --- | --- | --- | --- | --- |
| CPV2655-F | CCAGATCATCCATCAACATCA | 2655-2675 | 857 bp | 35/55 ◦C | Decaro et al., 2008 |
| CPV3511-R | TGAACATCATCTGGATCTGTACC | 3489-3511 |  |  |  |
| CPV3381-F | CCATGGAAACCAACCATACC | 3381-3400 | 717 bp |  |  |
| CPV4116-R | AGTTAATTCCTGTTTTACCTCCAA | 4093-4116 |  |  |  |
| 555for | CAGGAAGATATCCAGAAGGA | 4003-4022 | 583 bp |  |  |
| 555rev | GGTGCTAGTTGATATGTAATAAACA | 4561-4585 |  |  |  |

**Data regarding primers (names, sequences, and positions), expected amplicon size, and the number of amplification cycles and annealing temperature of the PCR methodologies used in this study**

ᵃ The genomic positions of probe/primers are given according to the prototype strain “FPV-b” (GenBank acc. no. M38246)

References

Decaro, N., Desario, C., Miccolupo, A., Campolo, M., Parisi, A., Martella, V., Amorisco, F., Lucente, M.S., Lavazza, A., Buonavoglia, C., 2008. Genetic analysis of feline panleukopenia viruses from cats with gastroenteritis. J. Gen. Virol. 89, 2290-2298. <https://doi.org/10.1099/vir.0.2008/001503-0>.
