# Supplementary material for: Emergence, surge, and fading of the novel feline parvovirus Thr390Ala mutant in Egyptian cats during 2023: insights from a comprehensive full-length VP2 genetic analysis
Source: BMC Vet Res. 2025 Oct 3;21:570. doi: 10.1186/s12917-025-05004-3 (PMC12492670; doi:10.1186/s12917-025-05004-3)
Supplement: Supplementary file 12 — Supplementary Material 12. [file 12917_2025_5004_MOESM12_ESM.docx]

**Supplementary Table 7**

**Data regarding reference feline parvovirus strains of canine origin**

| Strain name | Country | Date | FPV group | Isolation source | GenBank acc. no. | Age | Health status | Reference |
| --- | --- | --- | --- | --- | --- | --- | --- | --- |
| 5371 | Australia | 2019 | G2 | Not provided | MZ362883 | Not reported | Not reported | Unpublished |
| C-DY6 | China | 2019 | G1 | Stool | OK128324 | Not reported | Not reported | Unpublished |
| JSYZ-85 | China | 2019 | G1 | Stool | MW017596 | 5 months | High fever, diarrhea, and dehydration | Wen et al., 2024 |
| C-F9 | China | 2019 | G3 | Stool | MZ913315 | Not reported | Not reported | Unpublished |
| C-F88 | China | 2019 | G3 | Stool | MZ913316 | Not reported | Not reported | Unpublished |
| C-1 | China | 2019 | G3 | Stool | MZ913317 | Not reported | Not reported | Unpublished |
| C-4 | China | 2019 | G3 | Stool | MZ913318 | Not reported | Not reported | Unpublished |
| C-9 | China | 2019 | G3 | Stool | MZ913319 | Not reported | Not reported | Unpublished |
| ZJHN-135 | China | 2020 | G1 | Stool | MW017616 | 3 months | High fever, diarrhea, and dehydration | Wen et al., 2024 |
| ZJHN-138 | China | 2020 | G1 | Stool | MW017618 | 1 month | High fever and vomiting | Wen et al., 2024 |
| D7 | China | 2021 | G1 | Stool | OK128325 | Not reported | Not reported | Unpublished |
| D13 | China | 2021 | G3 | Stool | MZ913314 | Not reported | Not reported | Unpublished |
| JSZJ1 | China | 2022 | G1 | Not provided | OR194110 | Not reported | Not reported | Unpublished |
| JSZJ2 | China | 2022 | G1 | Not provided | OR194111 | Not reported | Not reported | Unpublished |
| LZ05 | China | 2022 | G1 | Not provided | OQ869254 | Not reported | Not reported | Unpublished |
| 139-188 | Egypt | 2021 | G3 | Blood | OM638043 | 1 year | Fever of unknown origin | Diakoudi et al., 2022 |
| 164-1 | Italy | 2021 | G3 | Stool | OM638042 | Adult | severe gastrointestinal symptoms | Diakoudi et al., 2022 |
| 18Q234-1 | Korea | 2018 | G3 | Spleen | MW035310 | Not reported | Not reported | Unpublished |
| 19SP_CK-8 | Korea | 2019 | G3 | Stool | MW035309 | Not reported | Not reported | Unpublished |
| HN39AA | Vietnam | 2017 | G3 | Stool | MK357738 | 5 Months | Diarrhea, Vomiting | Hoang et al., 2020 |
| HN3 | Vietnam | 2017 | G3 | Stool | MK357739 | 3 Months | Diarrhea, Vomiting | Hoang et al., 2020 |
| HN40AA | Vietnam | 2017 | G3 | Stool | MK357740 | 4 Months | Diarrhea, Vomiting | Hoang et al., 2020 |
| HN10 | Vietnam | 2018 | G3 | Stool | MK357741 | 2 Months | Diarrhea, Vomiting | Hoang et al., 2020 |
| HN41AA | Vietnam | 2018 | G3 | Stool | MK357742 | 9 Months | Diarrhea, Vomiting | Hoang et al., 2020 |
| HN7 | Vietnam | 2018 | G3 | Stool | MK357743 | 3 Months | Diarrhea, Vomiting | Hoang et al., 2020 |

References

Diakoudi, G., Desario, C., Lanave, G., Salucci, S., Ndiana, L. A., Zarea, A.A.K., Fouad, E.A., Lorusso, A., Alfano, F., Cavalli, A., Buonavoglia, C., Martella, V., Decaro, N., 2022. Feline Panleukopenia Virus in Dogs from Italy and Egypt. Emerg. Infect. Dis. 28, 1933–1935. <https://doi.org/10.3201/eid2809.220388>

Hoang, M., Wu, C.N., Lin, C.F., Nguyen, H.T.T., Le, V.P., Chiou, M.T., Lin, C.N., 2020. Genetic characterization of feline panleukopenia virus from dogs in Vietnam reveals a unique Thr101 mutation in VP2. PeerJ. 8, e9752. <https://doi.org/10.7717/peerj.9752>.

Wen, Y., Tang, Z., Wang, K., Geng, Z., Yang, S., Guo, J., Chen, Y., Wang, J., Fan, Z., Chen, P., Qian, J., 2024. Epidemiological and Molecular Investigation of Feline Panleukopenia Virus Infection in China. Viruses 16, 1967. <https://doi.org/10.3390/v16121967>
